# Variations in photoreceptor throughput to mouse visual cortex and the unique effects on tuning

**DOI:** 10.1038/s41598-021-90650-4

**Published:** 2021-06-07

**Authors:** I. Rhim, G. Coello-Reyes, I. Nauhaus

**Affiliations:** 1 Department of Psychology, University of Texas At Austin, 108 E. Dean Keeton, Austin, TX 78712 USA; 2 Department of Neuroscience, University of Texas At Austin, 1 University Station, Stop C7000, Austin, TX 78712 USA; 3 Center for Perceptual Systems, University of Texas At Austin, 108 E. Dean Keeton, Austin, TX 78712 USA

**Keywords:** Visual system, Colour vision, Motion detection, Object vision, Pattern vision, Retina, Striate cortex

## Abstract

Visual input to primary visual cortex (V1) depends on highly adaptive filtering in the retina. In turn, isolation of V1 computations requires experimental control of retinal adaptation to infer its spatio-temporal-chromatic output. Here, we measure the balance of input to mouse V1, in the anesthetized setup, from the three main photoreceptor opsins—M-opsin, S-opsin, and rhodopsin—as a function of two stimulus dimensions. The first dimension is the level of light adaptation within the mesopic range, which governs the balance of rod and cone inputs to cortex. The second stimulus dimension is retinotopic position, which governs the balance of S- and M-cone opsin input due to the opsin expression gradient in the retina. The fitted model predicts opsin input under arbitrary lighting environments, which provides a much-needed handle on in-vivo studies of the mouse visual system. We use it here to reveal that V1 is rod-mediated in common laboratory settings yet cone-mediated in natural daylight. Next, we compare functional properties of V1 under rod and cone-mediated inputs. The results show that cone-mediated V1 responds to 2.5-fold higher temporal frequencies than rod-mediated V1. Furthermore, cone-mediated V1 has smaller receptive fields, yet similar spatial frequency tuning. V1 responses in rod-deficient (Gnat1^−/−^) mice confirm that the effects are due to differences in photoreceptor opsin contribution.

## Introduction

The visual system has a cascade of light adaptation mechanisms that accumulate to provide a wide dynamic range. Light adaptation in the retina is first built from differences in rod and cone sensitivity, followed by their respective downstream circuits. In each case, sensitivity increases with a drop in ambient light, which is traded for spatio-temporal-chromatic resolution. That is, monochromatic rods are sensitive to minute changes at the lowest light levels, which are sent through pathways that integrate over a broader window of space and time than cones^1–4^. In turn, rods and cones set the stage for subsequent branching of parallel pathways that remain relevant to information processing in the rest of the visual system^5^. Quantifying downstream transformations requires knowledge of retinal output, and thus rod vs. cone contributions for a given visual stimulus.

Unfortunately, most cortical studies in the mouse are based on varying and unknown degrees of input from rods vs. cones. The tendency has been to adopt a similar average luminance (~40 cd/m^2^) as that used in classic studies with larger mammals and commercial displays. On the one hand, there are reasons to believe a similar luminance will place the rodent visual system in a photopic regime, which is based on the anatomy and in-vitro physiology of the retina. The mouse retina has in place the major architectural hallmarks of the primate retina^6^. Notably, this includes a substantial majority (> 95%) of rods in the photoreceptor mosaic, which then feed into rod bipolar and AII amacrine cells^7^. On the other hand, there are reasons why primate models of photopic threshold may not be accurate in the rodent. Many primate studies are done near the fovea where the proportion of cones is much higher. Also, the mouse’s eye and pupil are much smaller, which affect retinal irradiance, and thus rod saturation for a given lighting environment. Another species difference is rod sensitivity to UV wavelengths, which limits their sensitivity to commercial displays . Adding to the challenge of cross-species comparison, most retinal studies on rod saturation performed in-vitro do not include the optics of the eye and the pigment epithelium, making it more difficult to extrapolate to the in-vivo preparation.

Here, we measured V1 responses in anesthetized mice as a function of graded changes in rod saturation. Relative to commercial displays, our display generated much higher rod isomerization rates, which are uniform across viewing angles. Next, using a simple model that linearly combines the input from three photoreceptor opsins—rhodopsin, cone S-opsin, and cone M-opsin—we quantified the relative contributions of each as a function of photoisomerizations(R*)/rod/sec and retinotopic location. First, the model predicts that the dimmest and brightest settings yield 75% and 5% rod input, relative to cones. The model also gives the balance of cone M- and S-opsin input as a function vertical retinotopy. Finally, we used the model to predict the state of retinal adaptation under two hypothetical lighting conditions: a commercial display and outdoor lighting in an urban setting^8^. We predict that although a dilated pupil does not allow for rod saturation with a commercial display, mouse rods quickly saturate at the onset of sunrise. Finally, we isolated cone and rod contrast to characterize their respective spatio-temporal properties in V1 with 2-photon imaging. We showed that spatial tuning changes little between rod- and cone-mediated vision. However, cone-mediated V1 encoded substantially higher temporal frequency than rods.

## Results

### Measurements of photoreceptor contributions to mouse V1

**Figure 1:**
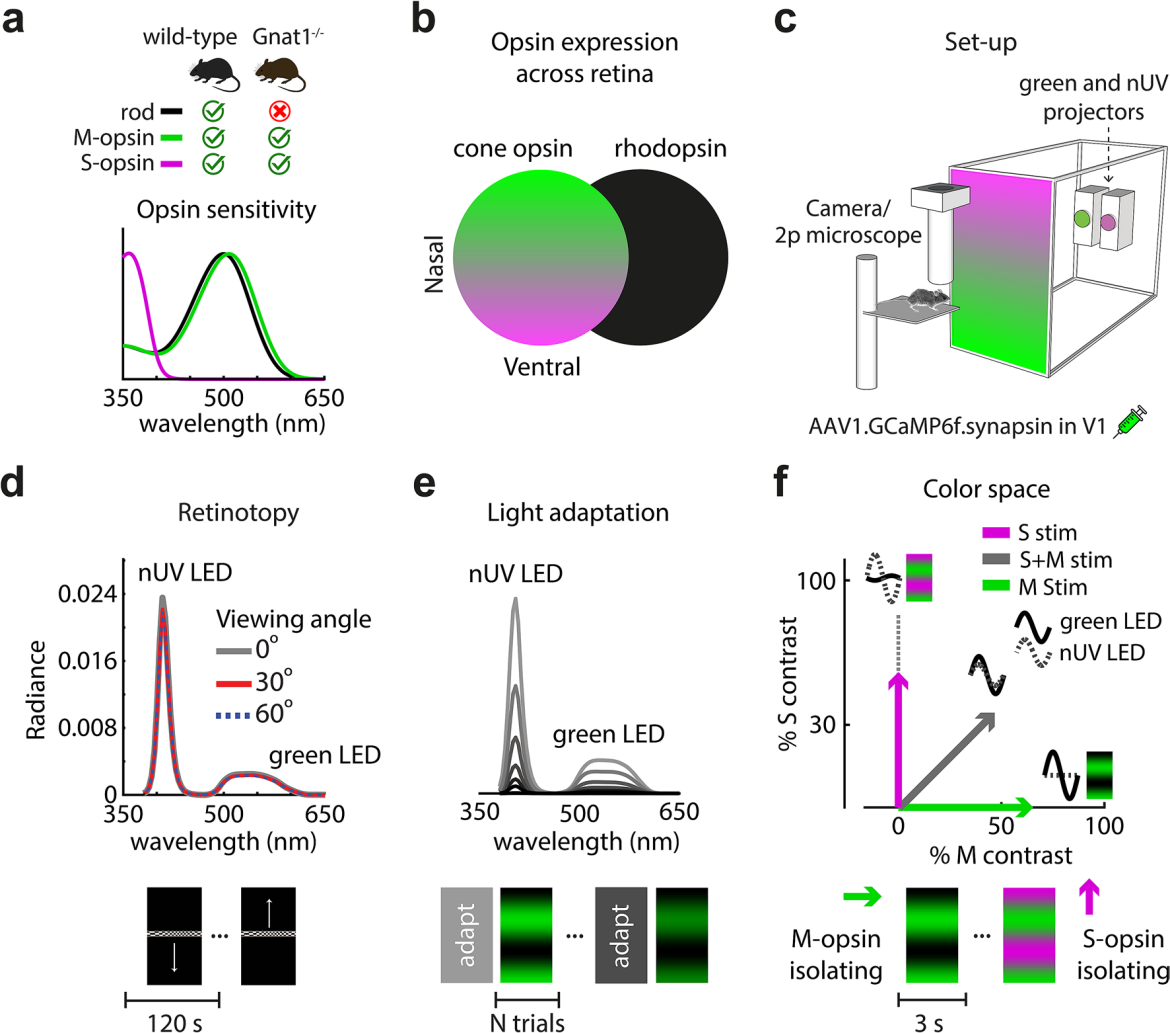
Imaging and visual stimuli setup for mapping retinotopy and color in the mouse V1. (**a**) Opsin sensitivity functions for 3 different types of opsins found in rod and cone photoreceptors. Rhodopsin peaks at 498 nm, while cone S-opsin and M-opsin peak at 360 nm and 508 nm, respectively. 2 mouse genotypes were used: wild-type and Gnat1^−/−^, which is a rod-deficient transgenic strain. (**b**) Illustration of cone S/M-opsin expression gradient in mouse retina (left) versus rhodopsin expression (right). Ventral retina (upper visual field) expresses predominantly S-opsin while dorsal retina (lower visual field) expresses predominantly M-opsin. Rods are uniformly expressed throughout retina. (**c**) Rear projection of visual stimuli using two monochromatic projectors, near-UV (nUV) and green. Screen color depicts corresponding S/M-opsin expression in mouse’s retina. (**d**) Measures of the display’s spectral radiance at three viewing angles. Uniformity is required for controlled light adaptation across the retinotopy. Below are the stimuli used to map retinotopy. (**e**) A total of 6 background light levels were used to adapt the retina, with each level scaling the spectral radiance by a factor of 2. Below is an illustration of the adaptation paradigm. Prior to a block of test trials with drifting gratings, the retina was adapted for 10 min by the midpoint (i.e. “gray level”) of the test trials. (**f**) Drifting gratings were calibrated to oscillate along one of three axes in S and M cone-opsin space: S, M, and S + M. The sine-wave insets indicate relative green and nUV LED amplitude and phase used for each of the three color axes. Below is an illustration of a stimulus paradigm for measuring color preference, where each trial showed a different color at a given adaptation level.

We imaged anesthetized mouse V1 responses to visual stimuli that were calibrated to modulate distinct photoreceptor opsins across the retina. Two-photon and widefield calcium imaging of neurons was performed after viral-mediated expression of GCaMP6f^9^. Figure 1 outlines features of the mouse preparation and visual stimulus paradigm that were common to all results from this study. Most recordings were done in wild-type (WT) mice, and a subset of experiments was repeated in mice lacking rod function (Gnat1^−/−^)^10^. The WT mouse retina has 3 main photoreceptor opsins—cone M-opsin, cone S-opsin, and rod rhodopsin—which have unique spectral sensitivities functions (Fig. 1a) and spatial distributions across the retina (Fig. 1b). The upper region of the visual field is filtered primarily through the S-opsin and rhodopsin sensitivity functions. Conversely, in the lower extreme of the visual field, images are filtered through the M-opsin and rhodopsin sensitivity functions^4^, which are very similar.

A major goal of the study was to quantify the balance of input to V1 from all three opsins as a function of visual field location and light adaptation. To this end, visual stimuli systematically varied along 3 dimensions—retinotopy, background light level, and color. The vertical retinotopy was mapped at each pixel (widefield imaging) or neuron (2-photon imaging) based on responses to a monochromatic vertical drifting bar^11^. The display’s emitted spectral power (radiance) was nearly constant across the range of viewing angles under study (+ /- 60°), which allowed for controlled measurements of light adaptation across the retinotopy (Fig. 1d). To alter the state of light adaptation, the display’s spectral power was scaled to a new midpoint for 10 min, followed by a continuous block of full-field drifting gratings that maintained the same midpoint (Fig. 1e). As will be shown, varying the state of light adaptation with our display allowed for a wide dynamic range of rod saturation.

Within each block of light adaptation, drifting gratings were shown at variable orientation to generate robust V1 responses along isolated axes of color space, and/or along a range of spatio-temporal frequencies. Initially, we show results where spatio-temporal frequency is kept constant and responses are compared between M and S cone-opsin contrasts (Fig. 1f). These measures of M/S color tuning vs. light adaptation are used to model a) rod vs. cone inputs to V1 as a function of rod photoisomerization rates, along with b) the retinotopic distribution of pure cone-opsin inputs to V1. At the end, we use a related paradigm to compare rod-mediated and cone-mediated spatio-temporal tuning properties in V1.

### Revealing the cone mosaic via increasing rod saturation

**Figure 2:**
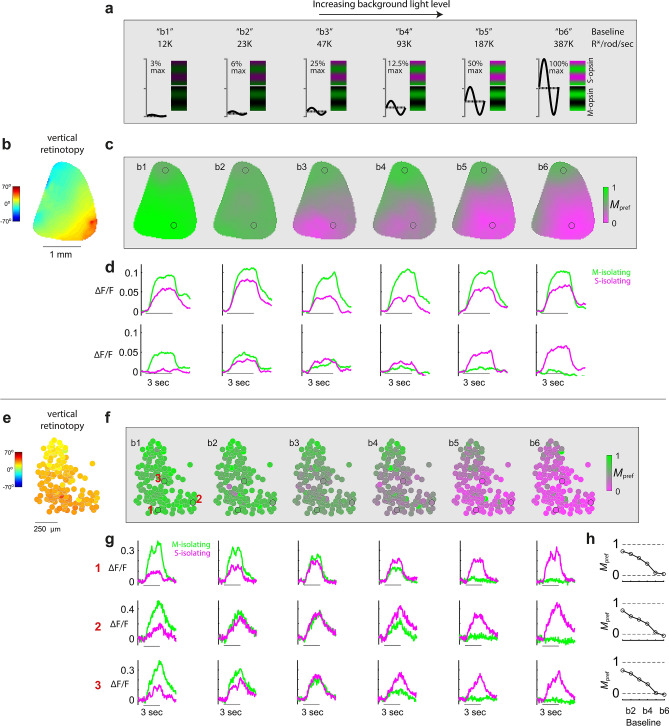
Revealing the cone mosaic’s M-to-S gradient in V1 via progressive rod saturation. (**a**) Illustration of the stimuli used to measure color tuning across 6 states of background light adaptation, “b1-b6”. Each subsequent adaptation levels have mean gray values that differ by a factor of 2, and constant sinewave contrast, depicted on left side of each subpanel. Prior to each level, the eye was adapted at the same gray value for 10 min. Each trial within a level block had either a S-opsin or M-opsin isolating grating, depicted on right side of each subpanel. (**b–d**) Wide-field calcium imaging. (**b**) Map of V1’s vertical retinotopy. (**c**) Maps of *M*_*pref*_, at each of the 6 states of light adaptation. *M*_*pref*_ at each pixel indicates the preference for the M-opsin color axis, over the S-opsin color axis. At low light levels, *M*_*pref*_ ~ 1 at all pixels, indicating rod input—rod inputs are uniform and have nearly identical spectral sensitivity as M-ospin. At higher light levels, rods saturate, revealing the M-to-S cone opsin gradient. (**d**) Two rows show time courses of fluorescence signal at the 2 ROI’s indicated in the map. Top circle corresponds to lower visual field; bottom circle corresponds to upper visual field. Green and violet traces are responses to M-opsin and S-opsin gratings, respectively. The line under each pair of traces indicates the stimulus duration. (**e–h**) 2-photon calcium imaging. (**e**) V1 map of vertical retinotopy, where each circle is the location of an example neuron. (**f**) Maps of *M*_*pref*_, at 6 states of light adaptation. (**g**) Each row of time courses is from a different neuron in the ROI (see their locations in *M*_*pref*_ map at left). Green and violet traces are responses to M-opsin and S-opsin gratings, respectively. (**h**) Right-most column shows each example neuron’s mean *M*_*pref*_ during stimulus presentation, as a function of light level.

Here we describe results from the visual stimulus paradigm introduced above, which varies color and light adaptation. This analysis is limited to experiments with an artificially dilated pupil; subsequent experiments will expand our findings to understand the impact of an undilated pupil. Since rods are monochromatic and uniformly distributed across the mouse retina, the entire visual field at night is filtered through a common spectral sensitivity function. However, when the dichromatic cone population is allowed to take over, there is a gradient of spectral sensitivity along the retina’s dorsoventral axis that is conveyed to V1 and higher visual areas^12^. What are the dynamics of the monochromatic-to-dichromatic photoreceptor transformation at the level of V1 topography, and does our current setup allow for it to be resolved and thus modeled? To address these questions, we used the experimental paradigm shown in Fig. 2a; S- and M-isolating gratings of equal contrast were presented after adapting to 6 different light levels.

For each neuron from the 2-photon imaging, and pixel from widefield imaging, we calculated a metric for color preference, *M*_pref_ = *F*_*M*_*/*(*F*_*S*_ + *F*_*M*_), where *F*_*M*_ and *F*_*S*_ are the mean fluorescence changes in response to the M- and S-isolating stimuli, respectively. Figure 2 shows the graded unveiling of the *M*_pref_ map. At the lowest light levels, neurons across the entire V1 retinotopy were mostly responsive to M-isolating gratings, indicating a predominance of rod input. Increasing light levels induced a graded *exchange* in responsiveness: responses to M-isolating gratings fell, and responses to S-isolating gratings rose (Fig. 2d,g). In the upper visual field, the exchange is especially dramatic, as this part of the cone mosaic is dominated by S-opsin—neurons go from strong preference for the M-opsin gratings (*M*_pref_ ~ 1) to strong preference for S-opsin gratings (*M*_pref_ ~ 0) with increasing light level. These examples confirm that the display generates a sufficient range of both intensity and color to model photoreceptor contributions across the V1 topography.

### Modeling opsin contributions to V1 as a function of isomerization rates and visual field location

Next, using the visual stimulus paradigm shown in Fig. 2a, the two-photon data was combined across animals to model a) the balance of rod and cone inputs as a function of light level and b) the balance of S vs. M cone-opsin inputs as a function of retinotopy.

#### Model description

Here, we summarize key elements of the model used to quantify photoreceptor contributions to V1. The equations given in the Methods describe the response of a V1 neuron to each chromatic grating (S and M-opsin isolating ) as a linear combination of 3 inputs: rods, S-opsin, and M-opsin. The magnitude of rod inputs depends only on light levels (not retinotopy), whereas the magnitude of S and M cone opsin inputs depends only on location along the vertical retinotopy (not light level). One assumption with this model is that rod inputs are uniform across the visual field in an animal that lacks a fovea^7^. A second assumption is that cone responses maintain Weber adaptation, meaning that responses depend on contrast and not the range of background light levels used in this study. We tested this latter assumption, given the proximity of our lowest light level to cone thresholds (i.e. their “dark light”) established in prior studies^13,14^.

**Figure 3:**
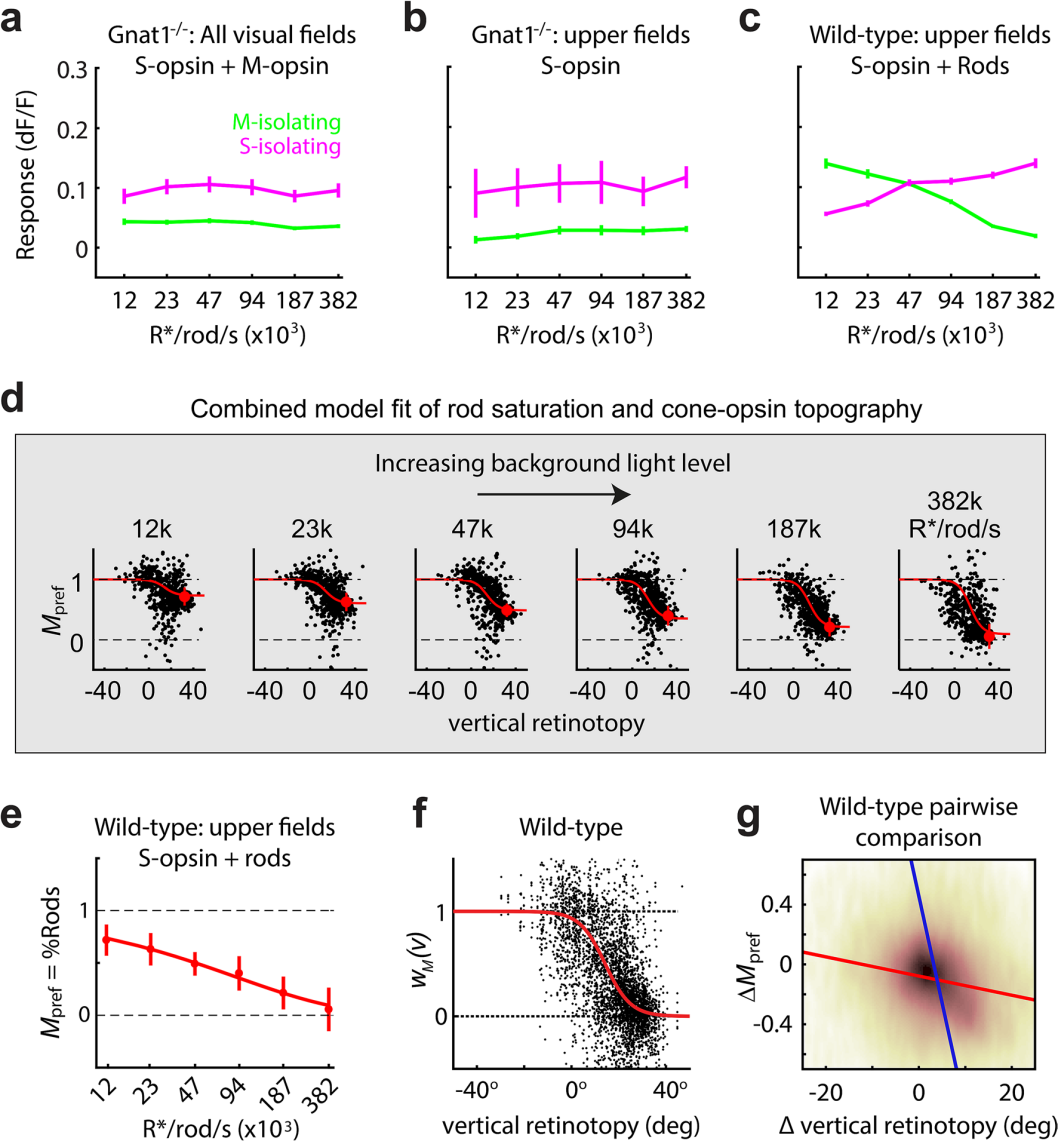
Modeling photoreceptor inputs as a function of light adaptation and vertical retinotopy. (**a**) Mean and standard error responses of V1 neurons in Gnat1^−/−^ mice, at 6 light levels. This was used to confirm that cones are in a constant state of Weber adaptation across the range of light levels. (**b**) The same analysis was performed as in ‘a’ using a subset of the data, upper visual field neurons (> 30°), which limits the cone inputs to S-opsin. (**c**) Mean and standard error responses of V1 neurons in WT mice, at 6 light levels. Data was limited to the upper visual field to highlight the population’s “switch” in responsiveness from rod- to cone-mediated inputs. (**d**) The six panels of *M*_*pref*_ vs. vertical retinotopy show data points from the same WT population, at the six states of light adaptation. Overlayed in red line is the model fit of *M*_*pref*_ as a function of vertical retinotopy and light adaptation (see “Methods”). Red dots indicate population average for neurons in the upper visual field of WT mice, which are shown again in 'e'. (**e**) The color-tuning metric, “*M*_*pref*_”, when limited to neurons in the upper visual field of WT mice, is effectively a measure of the balance of rod vs. cone input, “%Rods”. The mean and standard error bars of %Rods are shown as a function of light adaptation, and the line shows the model fit. (**f**) The y-axis, “*w*_*m*_(*v*)”, is an estimate of the balance of M- and S- cone opsin providing input to V1 at each retinotopic location. 1 and 0 indicate 100% M- and S-opsin, respectively. Data points are an accumulation from all the panels in ‘d’, after normalizing by the model fit along the dimension of light intensity using the %Rod model in ‘e’. The red line is the model fit of the retina’s cone opsin gradient (M vs. S cone-opsin input) vs. vertical retinotopy, recapitulated in V1. (**g**) Population pairwise comparison of ∆*M*_*pref*_ as a function of ∆vertical retinotopy (r = -0.21; p < 10^−32^). For each pair of cells, difference in *M*_*pref*_ is plotted against difference in vertical retinotopy, resulting in a slope of -0.006 ∆*M*_pref_/∆retinotopy (red line), regressing over the change in vertical retinotopy, and a slope of -7.0 ∆retinotopy/∆*M*_pref_ (blue line) regressing over the change in *M*_pref_. Background brown heatmap indicates density of data points.

To test for Weber adaptation in cones, we measured V1 responses as a function of light level in mice with dysfunctional rods (Gnat1^−/−^)^10^, which showed that they do not increase with light level, for either S or M-opsin gratings (Fig. 3a). Figure 3a uses cells at all locations of the retinotopy, whereas Fig. 3b limits the population to neurons with receptive fields in the upper region of the visual field (> 30° above midline). In both cases, responses do not change significantly with light level (p > 0.05; Pearson correlation). Figure 3b also shows that neurons in the upper visual field have near zero response to the M-opsin gratings, as expected. The assumption of Weber adaptation for cones simplified the model, yet accounts for a wide range of lighting conditions. In the Methods, we derive the model in Eq. 5 below, which expresses the measurement of color tuning, *M*_pref_, as a function of the input from each opsin, at each retinotopic location and backround light level. 

5 $$M_{{{\text{pref}}}} \left( {b,v} \right) = 1 - \frac{{w_{s} \left( v \right)s_{s} }}{{s_{s} + w_{r} \left( b \right)r_{m} }}\quad {\text{Model of color tuning response}}$$

The two functions we solved for are $w_{r} \left( b \right)$ and $w_{s} \left( v \right)$.${ }w_{r} \left( b \right)$ is the unitless weight of rod input as a function of background light level. $w_{s} \left( v \right)$ is the unitless weight of S-opsin input as a function of vertical retinotopy. The corresponding weight of M-opsin input is $w_{m} \left( v \right) = 1 - w_{s} \left( v \right)$. $s_{s}$ is the S-opsin contrast of S-opsin gratings (~ 0.6), and $r_{m}$ is the rod contrast of the M-opsin gratings (~ 0.6). Under complete rod saturation, $w_{r} \left( b \right)$ approaches zero, giving $M_{{{\text{pref}}}} \left( {b,v} \right)\sim 1 - w_{s} \left( v \right) = w_{m} \left( v \right)$, which is the pure cone opsin retinotopic gradient. Oppositely, under low light levels, $w_{s} \left( v \right) \ll w_{r} \left( b \right)$, giving $M_{{{\text{pref}}}} \left( {b,v} \right)\sim$ 1, which is pure rod input that only responds to the M-opsin gratings across the entire V1 topography. Below, we describe the results of fitting $w_{s} \left( v \right)$ and $w_{r} \left( b \right)$, based on the measurements of $M_{{{\text{pref}}}} \left( {b,v} \right)$.

#### Model fit of rod saturation

To fit the model to data, we used two-photon responses from 11 WT mice. Figure 3d plots *M*_pref_ against vertical retinotopy, at all 6 light levels. The population shows graded rod saturation and the emerging cone mosaic with increasing light levels—i.e. from left-to-right, *M*_pref_ is reduced and becomes more steeply correlated with the retinotopy. To simplify the relationship between rod saturation and *M*_pref_, we limited the data to the subpopulation of neurons that represent the upper visual field (> 30°), which limits the photoreceptor opsin input to cone S-opsin and rods. Studies in the mouse retina show that cone signals in the upper visual field are almost exclusively mediated by S-opsin^4,15^—i.e. $w_{s} \left( {v > 30} \right) = 1$, where 30° is a conservative threshold based on these prior studies. By limiting responses to the upper field, it can be shown that the measured color preference, $M_{{{\text{pref}}}}$, is equal to the proportion of rod input, relative to cones, defined as %Rod(*b*) = $w_{r} \left( b \right)/\left[ {w_{r} \left( b \right) + w_{s} \left( {v > 30} \right){ }} \right]$. In turn, the y-axis of Fig. 3e is shown as “$M_{{{\text{pref}}}}$ = $\% \text{ Rod}$”. The trend of $\% \text{ Rod}\left( b \right)$ is well-fit by the equation $e^{{ - 0.072\left( b \right)^{0.586} }}$. The data shows that the minimum and maximum light level of the display (b1 and b6) yield rod contributions to V1 of 75% (%Rod = 0.75) and 5% (%Rod = 0.05), relative to the cones.

#### Model fit of M-to-S cone opsin topography in V1

In a previous study, we showed that the M-to-S cone opsin gradient is recapitulated in V1 and higher visual areas^12^. However, this prior study did not account for potential rod “contamination” in measuring the cone opsin map, and it employed intrinsic signal imaging. Here, we were able to remove the rod contamination using the model of %Rod(*b*) in Fig. 3e. The topographic map of cone opsin inputs to V1, $w_{m} \left( v \right) = 1 - w_{s} \left( v \right)$, is dependent on known variables in our model: $w_{m} \left( v \right) = 1 - \frac{{\left[ {1 - M_{{{\text{pref}}}} } \right]}}{{\left[ {1 - \% {\text{Rod}}} \right]}}.$ Specifically, this equation converts the measured values of color preference, $M_{{{\text{pref}}}} \left( {b,v} \right)$ (Fig. 3d, all panels), into the cone-opsin topographic map, $w_{m} \left( v \right)$ (Fig. 3f, black points), by way of the model fit to %Rod(b) (Fig. 3e). The fitted red curve in Fig. 3f is $\hat{w}_{m} \left( v \right) = \frac{1}{{1 + e^{{0.192\left( {v - 14} \right)}} }}$. Finally, $\hat{w}_{m} \left( v \right)$ was converted to $\hat{M}_{{{\text{pref}}}} \left( {b,v} \right)$, by way of the model fit to %Rod(*b*), and overlaid on the data points in Fig. 3d (red).

#### Pairwise comparison of M_pref_ against retinotopy in V1

Thus far, we’ve shown a clear relationship between the vertical retinotopy and color preference. To further quantify the cell-by-cell continuity of the maps, we compared the pairwise change in retinotopy to the pairwise change in $M_{{{\text{pref}}}}$. Baseline 5 in the WT population was used for this analysis (Fig. 3d, 187 k R*/rod/sec). For every pair of data points within each ROI (> 38 K pairs), we calculated the change in *M*_pref_ and in vertical retinotopy, which yielded the density plot in Fig. 3g. There is a significant correlation (*r* =  − 0.21; p < 10^−32^). Regressing over the change in vertical retinotopy gives a slope of − 0.006 (∆*M*_pref_/∆retinotopy), and regressing over the change in *M*_pref_ gives a slope of − 7.0 (∆retinotopy/∆*M*_pref_).

### A bright display does not yield cone-mediated V1 responses if the pupil is not fully dilated

**Figure 4:**
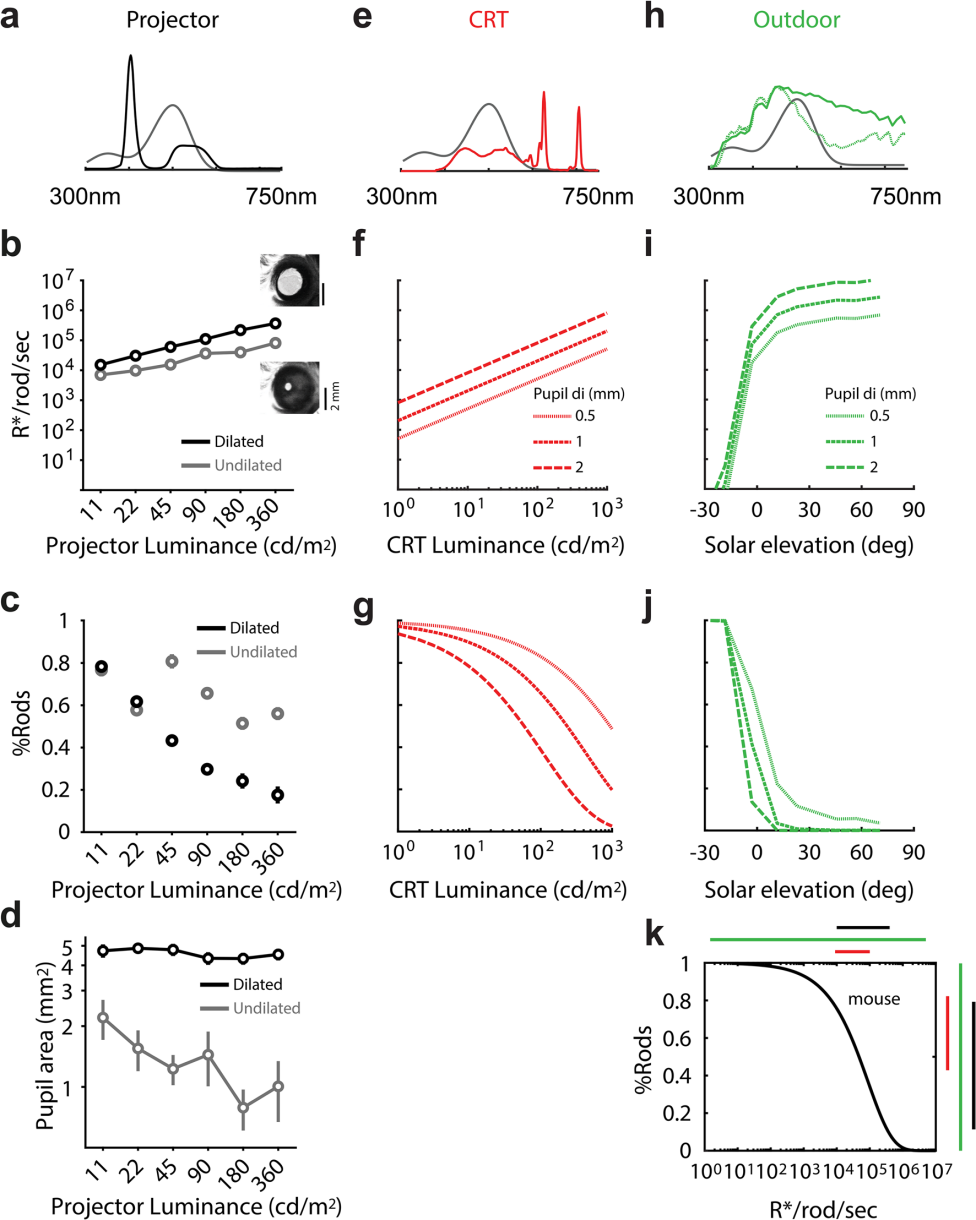
Variable states of retinal adaptation via pupil size, background light level, and stimulus source. (**a**) In black is the spectrum of the projector used in this study, with both green and nUV LEDs ‘”on”. (**b**) Rod isomerization rates measured at variable projector background light level, with and without application of tropicamide. To begin, the 6 adaptation blocks were shown, in ascending order (b1-to-b6), to an undilated (naive) pupil. Next, the pupil was dilated using tropicamide and reshown the 6 stimulus adaptation blocks in descending order (b6-to-b1). (**c**) %Rod input to V1 as a function of projector luminance values, for undilated and dilated pupils. (**d**) Mean and standard error of pupil area at 6 light levels (x-axis) with and without application of tropicamide. (**e**) In red is the spectral power distribution of a CRT with RGB guns all “on”. (f) Model of mouse rod isomerization rates over a wide range of CRT intensity, in cd/m^2^. (**g**) Prediction of %Rod contribution to V1 as a function of CRT luminance values. (**h**) In solid green is the power distribution of the CIE D65 standard, used as the daylight spectrum. The dashed green line is the spectrum used after sunset. Absolute daylight irradiance values were obtained from Spitschan et al.^8^ (**i**) Model of rod isomerization rates at varying solar elevation in an urban setting. (**j**) Prediction of %Rod contribution as a function of solar elevation. Positive and negative is above and below the horizon, respectively. (**k**) In black is the model fit of %Rod contribution vs. rod isomerization rates. It was used to transform the plots in ‘f’ and ‘i’ into the plots in ‘g’ and ‘j’. The color bars on top approximate the range of rod isomerization rates generated by each stimulus source. Similarly, the y-axis shows the ability of each source to push the rodent retina into a photopic (i.e. %Rod = 0) vs. scotopic (i.e. %Rod = 1) state.

Pupil size controls the irradiance at the retina, and thus the rod saturation for a given ambient light level. The measures of rod saturation in Figs. 2 and 3 were performed with an artificially dilated pupil and showed that we could reach ~ 95% cone-mediated input at our brightest setting. Next, we measured whether artificial dilation is a requirement for approaching rod saturation using our display (Fig. 4a), which is much brighter than more commonly used displays. We first swept through 6 levels of light adaptation (b1-b6) without artificial dilation, and then again with dilation using tropicamide (Fig. 4b). Without tropicamide, the pupil area was reduced by about twofold due to the 32-fold increase in light level. Following administration of tropicamide, the pupil area remained constant. We found that the mouse pupil diameter was about 2 mm when fully dilated (Fig. 4d), which is similar to measurements shown in previous studies^16,17^. Using pupil diameter measurements, light levels were converted into units of rod photoisomerization rates (R*/rod/sec) (Fig. 4b) (Methods). In each of the 12 blocks of trials, color tuning—viz. *M*_pref_—was measured. Just like Fig. 3e, we limited analyses to the upper visual field, where S-opsin dominates in cones, so that *M*_pref_ can be interpreted as %Rod input (Fig. 4c).

As expected from Fig. 3, there is a drop in %Rod input with increasing light level (Fig. 4c). A key difference between the dilated and undilated trends is the relative drop in *%*Rod at the highest light levels—they yield comparable *%*Rod near b1, but *%*Rod falls to much lower values at b6 in the dilated case. This clearly shows that our display cannot saturate rods through an undilated mouse pupil. Importantly, our display at b6 has far more rod isomerizing power than commercial displays. For a qualitative reference, most commercial displays have “luminance” values under 100 cd/m^2^, whereas the mean luminance of our green LED at b6 was 360 cd/m^2^. At the same time, luminance ignores the spectral power produced by the nUV LED in our set-up, which will also help saturate mouse rods. Specifically, the green LED and nUV LED account for 63% and 37% of the rod isomerizing power in the display based on their overlap with the rhodopsin sensitivity function, respectively.

### %Rod inputs to mouse V1 when exposed to outdoor lighting and commercial displays

In Fig. 3e, we used responses to variable light intensity to fit a model of the percentage of rod input to V1, relative to cone input, as a function of rod isomerization rate; viz. %Rod(*b*). Here, we used this model to make predictions of %Rod under two other relevant environments. We first asked what %Rod values are achieved under “standard” luminance (cd/m^2^) intensity produced by a commercial display. We show that these standard luminance levels yield mostly rod input. We then asked if the mouse retina can achieve a photopic state when exposed to natural outdoor lighting, and if so, what time of day the transition occurs.

Commercial displays do not drive cone S-opsin, but do drive rods and cone M-opsin. To make predictions of rod saturation relative to M-opsin inputs (i.e. %Rods) when exposed to a commercial display, we made radiometric measurements of a CRT monitor. The CRT results can be expected to generalize to other commercial displays since spectral irradiance profiles of ‘blue’ and ‘green’ will be quite similar (‘red’ may be unique in a CRT, but has minimal overlap with mouse photoreceptors). The CRT’s spectral radiance was compared to rod sensitivity functions (Fig. 4e) to calculate R*/rod/s as a function of CRT luminance (Fig. 4f). Then, each value of R*/rod/s was inserted into the model fit in Fig. 4k (previously shown with data in Fig. 3e) to yield the fraction of rod input, %Rods. Together, this gave curves of %Rods vs. luminance (Fig. 4g), which is a more relatable domain in most areas of vision science. A typical display produces luminance around 10^2^ cd/m^2^. At 10^2^ cd/m^2^, Fig. 4g shows that V1 is mostly rod-mediated unless the pupil is fully dilated. This differs from the primate in that 10^2^ cd/m^2^ is expected to put the retina into a photopic regime. Finally, we reiterate that these CRT predictions only pertain to M-opsin in the cone mosaic, which are expressed only in the dorsal half of the retina. The spectral power from commercial displays does not overlap with the spectral sensitivity function of S-opsin, so the cone mosaic will remain silent in the ventral half of the retina, regardless of background intensity.

If commercial displays cannot yield a cone-mediated mouse V1, a natural follow-up question is whether daylight can produce cone-mediated V1. For this, we used spectral irradiance measurements ${\text{(Watts}}/{\mu m}_{retina}^{2} /\Delta \lambda )$ at variable solar elevation taken in a recent study (Fig. 4h, green)^8^. Combined with the rod sensitivity function, the solar spectral irradiance measurements gave R*/rod/sec vs. solar elevation (Fig. 4i). Finally, we plugged R*/rod/sec into our model fit (Fig. 4k) to give %Rod vs. solar elevation (Fig. 4j). Unlike a commercial display, solar radiation has power at wavelengths to drive both M and S opsin. The curves show that the mouse retina rapidly approaches a photopic state after sunrise. Figure 4k provides a summary comparison of the dynamic range of light adaptation in the mouse retina for three lighting conditions. Natural outdoor lighting (green line) spans the full range, from scotopic to photopic. Both of the two experimental displays examined—our projector set-up and commercial displays—place the retina in the mesopic transition zone. However, the relatively incremental advantage provided by the nUV power and overall brightness in our projector system (Fig. 4k; compare blue and red, on top) is able to push the retina near a photopic state (Fig. 4k; compare blue and red, on right). In summary, although we have used seemingly extreme experimental condition to approach photopic vision, the difference from commercial displays is minor relative to the bounds of outdoor lighting.

A final note on interpretation of these results: the model of %Rod vs. rod isomerization rate is independent of cone adaptation under the assumption that cones are above the dark noise, and into Weber adaptation. It is for this reason that we only need to consider overlap with the rod sensitivity function to predict  %Rod at a given level of light adaptation. For instance, our nUV LED does not have much overlap with S-opsin sensitivity (Fig. 1a,d), yet has sufficient power to place the S-cones into Weber adaptation at the lowest light levels studied (Fig. 3a,b). In the case of the CRT, there is no overlap with S-opsin, so these plotted predictions only pertain to M-opsin in lower visual fields. In upper fields, the %Rod prediction is 100% with a commercial display.

### V1 responds to higher temporal frequencies when stimuli drive cones more than rods

Rods and cones are routed through different pathways in the retina that filter unique spatio-temporal properties of the visual scene for further processing in the cortex. To date, studies of detailed spatio-temporal tuning in mouse V1 are largely rod-mediated, as predicted by the results in Fig. 4. Here, we asked if V1’s spatio-temporal tuning properties change when we vary %Rods—the balance of rod and cone input. Two methods were used to alter %Rods—one that varied the state of light adaptation (i.e. rod saturation), and the other that varied color (i.e. rod and cone contrast). For the light adaptation method, rod-deficient (Gnat1^−/−^) mice were used as a control.

**Figure 5:**
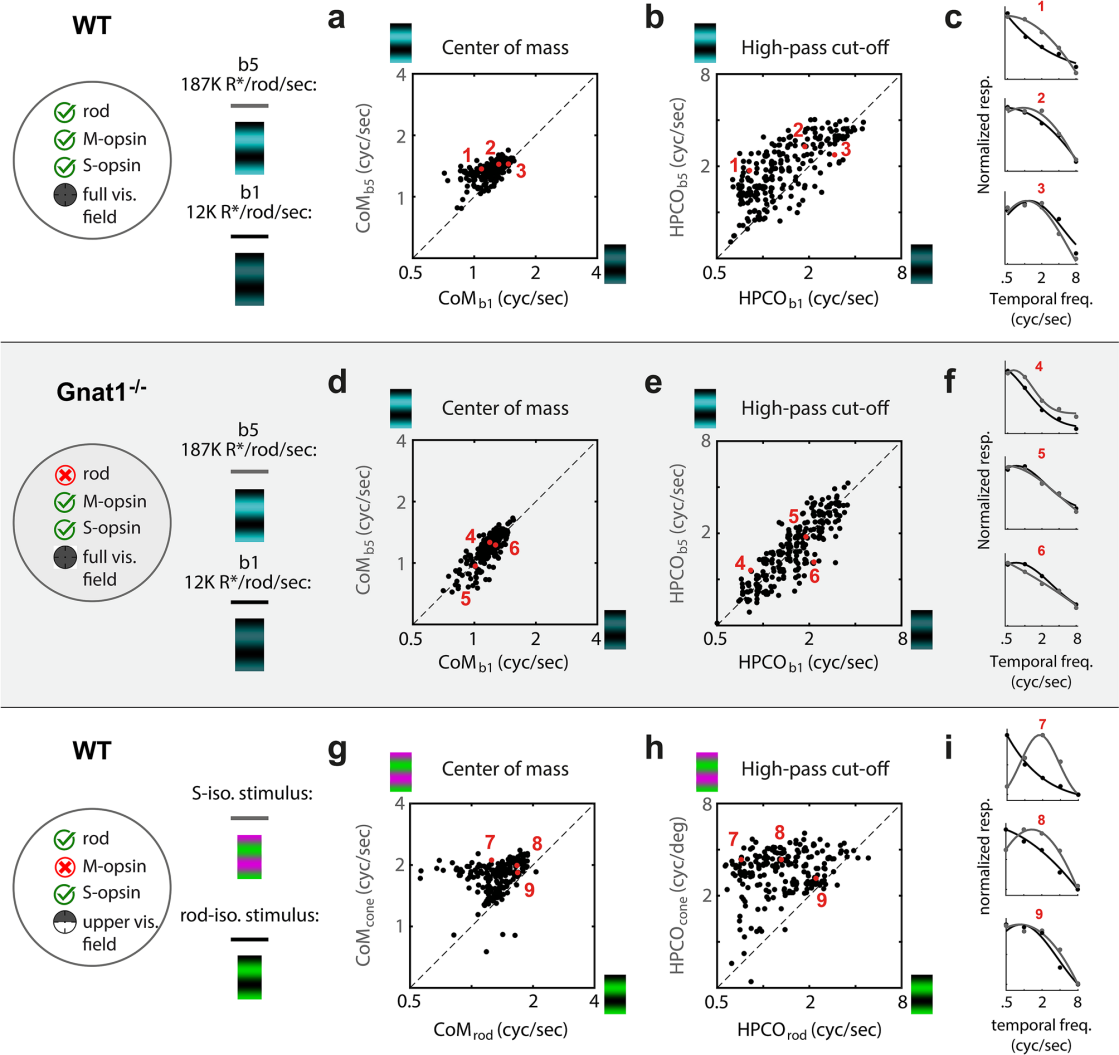
V1 responds to higher temporal frequencies when stimuli drive cones more than rods. Top row (‘a-c’) is data from WT mice, comparing temporal frequency tuning at two light adaptation levels, “b1” and “b5”, corresponding to 12 K and 187 K R*/rod/sec (n = 235). (**a**) Scatter plot compares the temporal frequency tuning curve’s center-of-mass (CoM) at b5 (y-axis) and b1 (x-axis). Unity line is dashed black line. The geometric mean of CoM_b5_/CoM_b1_ is 1.12 (i.e. 12% change; t-test: p = 2.47e^−35^). (**b**) Same as in ‘a’, but the temporal frequency tuning parameter is the high-pass cut-off frequency (HPCO). The geometric mean of HPCO_b5_/HPCO_b1_ is 1.38 (t-test: p = 1.76e^−32^). (**c**) Tuning and fits of 3 example neurons at the 5th, 50th, and 95th percentile of the HPCO_b5_/HPCO_b1_ distribution from ‘b’. Dots are normalized responses and lines are the fits. In each case, black and gray correspond to ‘b1’ and ‘b5’, respectively. Next, middle row of panels (‘d-f’) shows data from rod-deficient Gnat1^−/−^ mice, using the same experiment and analysis as in the top row (n = 244). (**d**) The geometric mean of CoM_b5_/CoM_b1_ is 1.00 (t-test: p = 0.012). (**e**) The geometric mean of HPCO_b5_/HPCO_b1_ is 1.01 (t-test: p = 0.0047). (**f**) Tuning and fits of 3 example neurons at the 5th, 50th, and 95th percentile of the distribution of HPCO_b5_/HPCO_b1_ from ‘e’. Bottom row of panels (‘g-i’) shows data from WT mice, limited to neurons with a receptive field 30-deg above the retina’s midline where opsin expression is mostly limited to S-opsin and rhodopsin (n = 218). Here, background light levels were held constant at b5 and tuning was compared between stimuli of isolating cone- and rod-contrast. (**g**) Scatter plot compares temporal frequency tuning CoM between the rod (x-axis) and cone (y-axis) isolating contrasts. Geometric mean of CoM_cone_/CoM_rod_ is 1.42 (t-test: p = 1.91e^−31^). (**h**) Same as in ‘g’, but the parameter measurement for each neuron and light level is HPCO. Geometric mean of HPCO_cone_/HPCO_rod_ is 2.54 (t-test: p = 1.97e^−51^). (**i**) Tuning and fits of 3 example neurons at the 5th, 50th, and 95th percentile of the distribution of HPCO_cone_/HPCO_rod_.

Temporal frequency tuning was measured under two background light levels, b1 (12 K R*/rod/sec) and b5 (200 K R*/rod/sec), where the predicted %Rod input is 75% (i.e. 25% cones) and 20% (i.e. 80% cones), respectively. Figure 5a-c and Table 1 show that there is a shift in tuning toward higher temporal frequencies when cones provide the primary input. For each neuron, we used two parameters from Gaussian fits to quantify tuning at each level of rod saturation, center-of-mass (CoM_b1_ & CoM_b5_) and the high-pass cut-off frequency (HPCO_b1_ & HPCO_b5_). To quantify differences, we calculated the geometric mean of the ratio between tuning parameters. In turn, paired t-tests were performed on the logs—e.g. log(CoM_b1_) vs. log(CoM_b5_). The geometric mean of CoM_b5_/CoM_b1_ was 1.12 (i.e. 12% change) and was significantly greater than one (p = 2.47e^−35^). The geometric mean of HPCO_b5_/HPCO_b1_ was 1.38 (p = 1.76e^−32^). To verify that these results were due to an exchange in activity between rods and cones, we repeated these experiments and analyses in Gnat1^−/−^ mice (Fig. 5d-f). While the difference in tuning for spatial or temporal frequencies between the two light levels were statistically significant, there were nominal difference in calculated means: geometric mean of CoM_b5_/CoM_b1_ = 1.00 (p = 0.012); geometric of HPCO_b5_/HPCO_b1_ = 1.01 (p = 0.0047).

**Table 1:** Summary of spatio-temporal tuning estimates, outlining parameter mean and standard deviation values from Figs. 5, 6 and 7. Parameter estimates for temporal frequency, spatial frequency, and receptive field size mapping experiments from Figs. 5, 6 and 7, respectively, compiled in table format. First 2 rows outline temporal frequency curve fit parameter estimates of center-of-mass and high-pass cutoff for “b1” (12 K R*/rod/sec) vs. “b5” (187 K R*/rod/sec) conditions in wild-type mice (3rd column), “b1” vs. “b5” in rodless (Gnat1^−/−^) mice (4th column), and “b5” cone vs. rod inputs in wild-type mice (5th column). “Rod” and “S-opsin” in last (5th) column indicate M-opsin contrast and S-opsin contrast stimuli used, respectively. Next 3 rows outline spatial frequency curve fit parameter estimates of center-of-mass, high-pass cutoff, and peak for “b1” vs. “b5” conditions in wild-type mice (3rd column), “b1” vs. “b5” but in rodless (Gnat1^−/−^) mice (4th column), and “b5” cone vs. rod inputs in wild-type mice (5th column). Last row outlines receptive field size comparison, measured as 2σ of Gaussian fit, between “b5” rod vs. cone inputs in wild-type mice. For each measurement listed, “*mu *(± *std*)” format is used to denote geometric mean value followed by standard deviation for temporal-spatial frequency parameters. “*mu *(± *std*)” format is used to denote arithmetic mean value followed by standard deviation for receptive field size estimates. “*/**/***” state statistical significance of p-values under .05, .01, and .001, respectively.

				
Temporal frequency (cyc/s)	Center-of-mass	b1: 1.19 (± .17) b5: 1.33 (± .13)***	b1: 1.30 (± .66) b5: 1.31 (± .81)*	Rod: 1.32 (± .31) S-opsin: 1.86 (± .24)***
	High-pass cutoff	b1: 1.38 (± .88) b5: 1.91 (± .84)***	b1: 1.95 (± 1.36) b5: 1.90 (± 1.35)**	Rod: 1.15 (± .68) S-opsin: 2.92 (± .88)***
Spatial frequency (cyc/deg)	Center-of-mass	b1: 0.05 (± .01) b5: 0.05 (± .013)***	b1: 0.050 (± .02) b5: 0.053 (± .018)*	Rod: 0.046 (± .018) S-opsin: 0.047 (± .013)***
	High-pass cutoff	b1: 0.084 (± .036) b5: 0.076 (± .022)***	b1: 0.085 (± .046) b5: 0.090 (± .041)*	Rod: 0.080 (± .038) S-opsin: 0.073 (± .026)***
	Peak	b1: 0.047 (± .024) b5: 0.045 (± .014)***	b1: 0.050 (± .033) b5: 0.053 (± .028)*	Rod: 0.043 (± .025) S-opsin: 0.040 (± .018)**
Receptive field size (deg)	Width	— —	— —	Rod: 35.62 (± 15.51) S-opsin: 27.74 (± 10.59)***

Next, we used a different method to compare temporal frequency tuning between rod and cone-mediated inputs. This method kept the light adaptation at a constant mesopic level, but varied rod and cone contrast. Just as in previously described experiments (Figs. 2, 3, 4), stimuli had color contrast that isolate either S-opsin or M-opsin (Fig. 1f). However, here the analysis was limited to neurons with upper receptive fields (> 30° above midline), so the M-opsin stimulus is effectively a rod-isolating stimulus; i.e. M-opsin and rods have nearly identical spectral sensitivity, yet the upper fields lack M-opsin. For the same reason, the S-opsin stimulus is effectively a cone-isolating stimulus (more specifically, the calculated rod contrast is 2% in response to the S-opsin stimulus). Figure 5g-i shows the comparison of temporal frequency tuning between the two different color directions. The results are consistent with the changes induced by different light adaptation levels in Fig. 5a-c, in that the cone-dominated tuning is shifted to higher values of temporal frequency. However, the differences are stronger with the method of rod- and cone-isolating contrasts, which may be attributed to purer separation of rod and cone drive. The geometric mean of CoM_cone_/CoM_rod_ is 1.42 (i.e. 42% change), and significantly greater than 1 (p = 1.91e^−31^). The geometric mean of HPCO_cone_/HPCO_rod_ exhibited the strongest differential, at 2.54 (p = 1.97e^−51^).

Many neurons in mouse V1 are highly selective for orientation. However, the results above used temporal frequency tuning curves from the average over orientation, as this gave the highest data yield using the criteria described in the methods. Averaging over orientation reduces the “signal”, but also reduces the “noise”. Nonetheless, this leaves open the possibility that results could differ when only the peak orientation is used. We thus repeated the analysis described immediately above (viz. for Fig. 5g-i), but only used the peak orientation to calculate temporal frequency tuning. Results were consistent, although the differential of HPCO was lower. The geometric mean of CoM_cone_/CoM_rod_ is 1.41, and significantly greater than 1 (p = 3.28e^−5^). The geometric mean of HPCO_cone_/HPCO_rod_ was 1.83 (p = 0.0012). Cell yield dropped to 68% of the original analysis method of averaging over orientation.

### Cone- and rod-mediated V1 responds to a similar band of spatial frequencies

**Figure 6:**
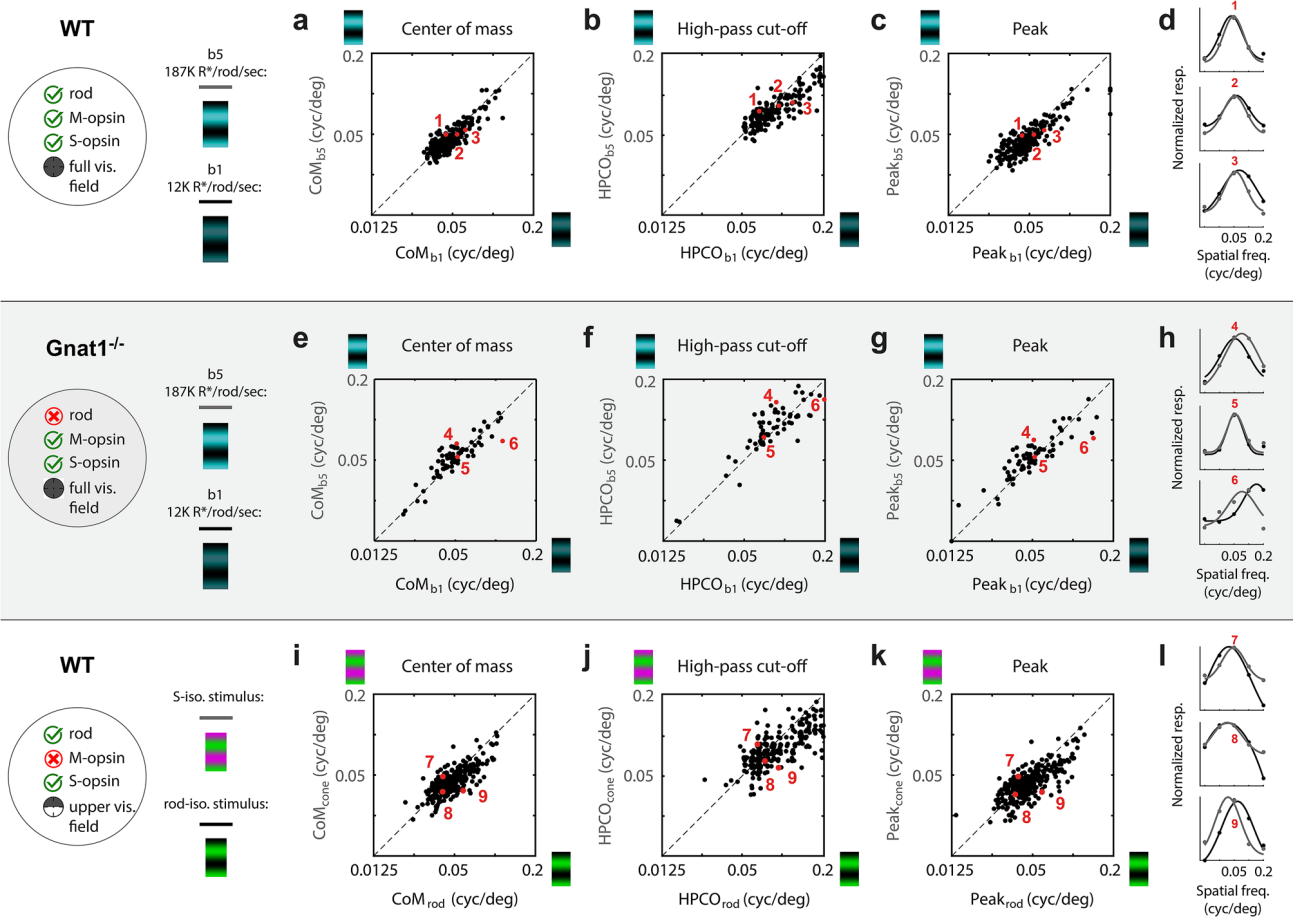
Rod-driven responses in V1 have marginally higher spatial frequency tuning than cone-driven responses. Top row (‘a-d’) shows data from WT mice, comparing spatial frequency tuning at two light adaptation levels, “b1” and “b5”, corresponding to 12 K and 187 K R*/rod/sec (n = 250). (**a**) Scatter plot compares center-of-mass (CoM) estimates of spatial frequency tuning curve at b5 (y-axis) and b1 (x-axis). Each data point represents a single neuron. Unity line is dashed black line. The geometric mean of CoM_b5_/CoM_b1_ is 0.96 (t-test: p = 1.04e^−6^). (**b**) Same as in ‘a’ but the spatial frequency tuning parameter is the high-pass cut-off frequency (HPCO). The geometric mean of HPCO_b5_/HPCO_b1_ is 0.90 (t-test: p = 2.40e^−17^). (**c**) Same as in ‘a,b’ but the spatial frequency tuning parameter is the peak spatial frequency (“Peak”). The geometric mean of Peak_b5_/Peak_b1_ is 0.94 (t-test: p = 2.07e^−7^). (**d**) Tuning and fits of 3 example neurons at the 5th, 50th, and 95th percentile of the distribution of HPCO_b5_/HPCO_b1_. Dots are normalized responses and lines are the fits. In each case, black and gray correspond to ‘b1’ and ‘b5’, respectively. Next, middle row of panels (‘e–h’) is data from rod-deficient Gnat1^−/−^ mice, using the same experiment and analysis as in the top row (n = 72). (**e**) The geometric mean of CoM_b5_/CoM_b1_ is 1.05 (t-test: p = .013). (**f**) The geometric mean HPCO_b5_/HPCO_b1_ is 1.06 (t-test: p = 0.021). (**g**) The geometric mean of Peak_b5_/Peak_b1_ is 1.06 (t-test: p = .029). (**h**) Tuning and fits of 3 example neurons at the 5th, 50th, and 95th percentile of the distribution of HPCO_b5_/HPCO_b1_ from ‘f’. Bottom row of panels (‘i-l’) shows data from WT mice, limited to neurons with a receptive field 30-deg above the midline where expression is mostly limited to S-opsin and rhodopsin (n = 361). Here, the background light levels were held constant at b4 or b5 and tuning was compared between stimuli of isolating cone- and rod-contrast. (**i**) Scatter plot compares spatial frequency tuning CoM between the rod (x-axis) and cone (y-axis) isolating contrasts. Geometric mean of CoM_cone_/CoM_rod_ is 1.02 (t-test: p = 3.35e^−4^). (**j**) Same as in ‘i’ but for HPCO. The geometric mean of HPCO_cone_/HPCO_rod_ is 0.91 (t-test: p = 1.79e^−4^). (**k**) Same as in ‘i’ but for Peak. The geometric mean of Peak_cone_/Peak_rod_ is 0.93 (t-test: p = 4.69e^−3^). (**l**) Spatial frequency tuning and fits of 3 example neurons at the 5th, 50th, and 95th percentile of the distribution of HPCO_cone_/HPCO_rod_.

Similar analyses were performed for spatial frequency tuning as those described above for temporal frequency tuning. Three parameters were taken from Gaussian fits to the spatial frequency tuning curves: peak location, CoM, and HPCO. The comparisons between two levels rod saturation (b1 and b5) in WT mice are shown in Fig. 6a-d and Table 1. The tuning is quite similar between the two states of adaptation. However, there is a small but significant shift in tuning to lower spatial frequencies when cones are the primary input: Geometric mean of Peak_b5_/Peak_b1_ = 0.94 (p = 2.07e^−7^), CoM_b5_/CoM_b1_ = 0.96 (p = 1.04^−6^), and HPCO_b5_/HPCO_b1_ = 0.90 (p = 2.4e^−17^). For Gnat1^−/−^ mice, this analysis also revealed a significant but minute difference in tuning for all of the three parameters. However, in this case, the higher light levels yielded tuning for higher spatial frequencies (Fig. 6e-h, Table 1).

Then, at a mesopic light level, we compared spatial frequency tuning between cone and rod stimuli in the upper visual field (> 30° above midline) (Fig. 6i-l). Similar to the results above with rod saturation, there was not much visually discernable difference in tuning across the two sets of gratings However, there was a statistically significant difference whereby rod mediated V1 responded to higher spatial frequencies. The main findings of the spatio-temporal tuning comparison between rod and cone inputs are summarized in Table 1.

Next, we repeated the analysis described immediately above (viz. for Fig. 6i-l), but calculated spatial frequency tuning at a single preferred orientation instead of averaging over all orientations. Results were consistent: Geometric mean of Peak_cone_/Peak_rod_ = 1.01 (p = 1.19e^−4^), CoM_cone_/CoM_rod_ = 0.99 (p = 0.0026), and HPCO_cone_/HPCO_rod_ = 0.93 (p = 0.0043). Similar to the temporal frequency analysis, when the data was reanalyzed using only the peak orientation, cell yield dropped to 42% of the original analysis method. 

### V1 has narrower receptive fields when stimuli drive cones more than rods

To further characterize rod vs. cone-mediated differences in V1’s spatial tuning, receptive field width was measured along the vertical dimension of the visual field. The contrast of horizontal bars against a mesopic background was along either the S- or M-axis of color space. As described above (Figs. 5g-i and 6i-l), under mesopic adaptation in the upper visual field, the S- and M-opsin stimuli isolate cones and rods, respectively.

**Figure 7:**
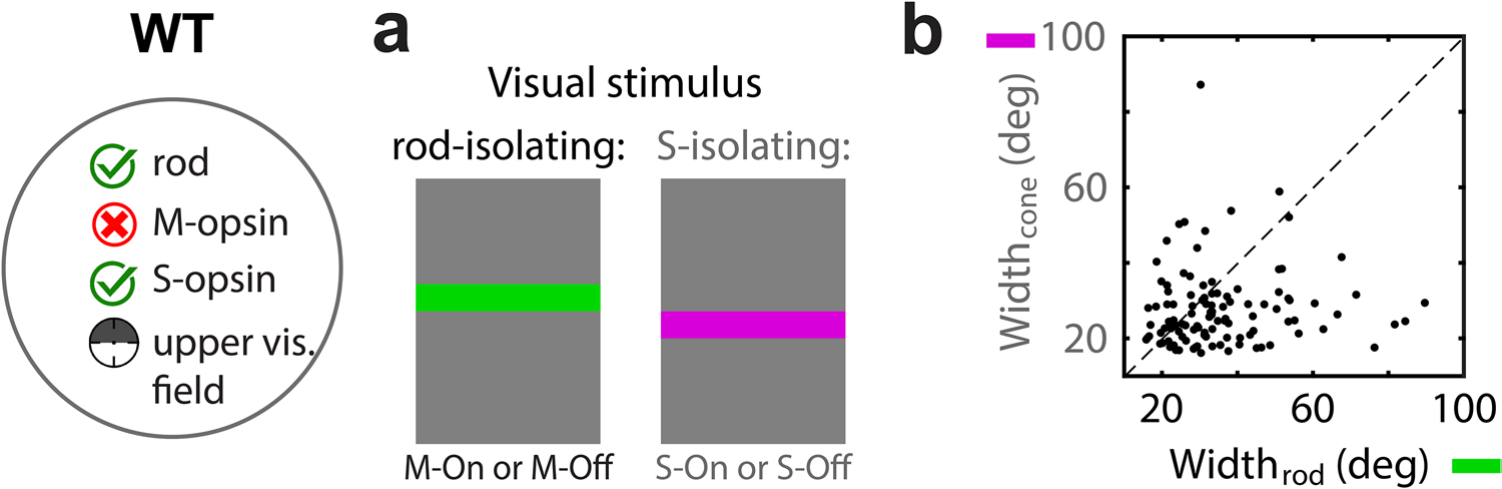
Rod-mediated V1 has larger receptive fields than cone-mediated V1. (**a**) Horizontal bars (6° in vertical width, at 2.8° intervals) were intermittently flashed on the screen, varying in color (M-ON, M-OFF, S-ON, or S-OFF) on constant background light level of ‘b5’ (187 K R*/rod/sec). Data is shown for WT mice, limited to neurons with a receptive field 30-deg above the midline where expression is mostly limited to S-opsin and rhodopsin (n = 108). A Gaussian curve is fit to each neuron’s tuning curve of response vs. bar position, for Rod and S-opsin contrast, which was used to compare receptive field size. Rod isolating contrasts combine responses from both M-ON and M-OFF bars. Cone isolating contrasts combine responses from both S-ON and S-OFF bars. (**b**) Scatter plot compares receptive field width (2σ) between rod (x-axis) and cone (y-axis) isolating contrasts. Each data point represents a single neuron. Unity line is dashed black line. The mean difference is 7.9 visual angle degrees; geometric mean of Width_cone_/Width_rod_ = 0.83 (t-test: 5.04e^−4^).

This yielded two spatial tuning curves for each neuron, to which the σ of Gaussian fits quantified the receptive field width under cone- and rod-mediated inputs (Fig. 7; Table 1). The mean receptive field width (2σ) for rod- and cone-mediated inputs was, 35.6° and 27.7°, respectively. The mean of the difference, Width_rod_–Width_cone_, was 7.9°. The geometric mean of their ratio, Width_cone_/Width_rod_, was 0.83 (p = 5.04e^−4^; t-test). In summary, V1 receptive fields are wider when rod-mediated.

## Discussion

The visual stimuli needed to drive cones in and around the fovea is well understood in the primate, which has guided the engineering of commercial displays that drive the perceptual gamut of trichromatic primate vision. The mouse retina, while exhibiting many similarities in structure and function to the primate, lacks a fovea and contains UV-sensitive cone-opsin. Furthermore, the geometry and optical properties of the mouse’s eye produce retinal irradiance for a given stimulus that will be different than in the primate eye^18^. Overall, the balance of inputs from rods and cones to the rest of the mouse’s visual system is difficult to extrapolate from primate psychophysics and rodent recordings in the retina. Here, we used a simple measure of color tuning to model the balance of rod and cone inputs to mouse V1, as a function of background light levels. With this model in-hand, we simulated the balance of rod and cone input to V1 when the eye is exposed to other relevant environments—sunrise puts the mouse retina into a photopic regime, whereas a commercial display does not. Finally, we used our calibration of photoreceptor inputs to design experiments that test the differential contributions of rods and cones to spatio-temporal tuning in V1. Using two different experimental methods, we show a significant increase in responsiveness to higher temporal frequencies in the transition from rod- to cone-mediated vision. We then used the same methods to show that cone-mediated vision produces narrower receptive fields, yet comparable spatial frequency tuning.

### Photoreceptor drive with commercial displays in prior studies of mouse visual cortex

Prior studies of the mouse visual cortex have largely ignored the photoreceptor classes being driven. Nevertheless, one can assume that visible wavelengths in the upper visual field produce rod-mediated responses. In the lower visual fields, the cones express green-sensitive M-opsin, and thus are capable of being driven by commercial displays. However, the simulation in Fig. 4g shows that the luminance values required to saturate the rods and achieve M-opsin mediated vision are outside the range of commercial displays, even with a fully dilated pupil.

An additional factor to consider with commercial displays is their attenuation of intensity with increasing viewing angle. This is an issue when imaging mouse visual cortex since a wider range of viewing angles is required to drive all the recorded cells across the retinotopy. Specifically, our simulation of %Rod for a commercial display (Fig. 4g) is expected to yield even higher values at the edges of common displays, assuming calibrations were made orthogonal to the screen. To quantify this, we made spectral radiance measurement of an LCD panel at variable viewing angle. It was found that peak radiance off the LCD panel was attenuated by 45% and 95% at 30° and 60° viewing angles, relative to the max at 0° viewing angle. As shown in Fig. 1d, the rear projection display used in this study is Lambertian—the spectral radiance does not change with viewing angle. In turn, our simulations on the required luminance to saturate rods with a commercial display (Fig. 4g) are effectively underestimating rod saturation at most screen positions.

Taken together, we believe that virtually all prior recordings in mouse visual cortex should be interpreted as rod-mediated, particularly those where a commercial display was used. As we show here, prior results on spatial frequency tuning may be expected to generalize to other light adaptation regimes, yet receptive field size and temporal frequency tuning are not. Even when UV light sources were used with non-commercial displays in prior studies, our data in Figs. 3e,4c shows that rod saturation is still quite unlikely. This would explain why a transition from green-to-UV selectivity, with increasing light intensity (e.g. as we show in Figs. 2 and 3), was not observed in^19^. Furthermore, studies that utilize combinations of UV and visible stimuli to assess downstream maps of S- and M-opsin selectivity are likely including rod input, thus obscuring the topographic S-to-M gradient^ 20,21^.

### The required light levels to reach cone-mediated responses can vary

The light intensity required to saturate rods has been measured at varying stages along the rodent visual pathway, and via medley techniques^4,13,22–24^, placing it somewhere between 10^3^ and 10^5^ R*/rod/sec. Although several factors are likely to contribute to this variability, a recent and notable study showed that a critical variable is the length of adaptation time^25^. After 10 min of exposure to 10^5^ R*/rod/sec, an intensity generally presumed to saturate rods, they showed that ~ 50% of ganglion cells regain their responsiveness. Our brightest light levels resulted in cone-mediated responses following a 10 min adaptation window, which may seem contradictory to these reported temporal dynamics of rod recovery. However, there are two distinctions worth noting.

First, our brightest light levels are just above 10^5^ R*/rod/sec, where rod saturation appears to be more sustainable^4,25,26^. Second, we calculated the balance of rods vs. cones (%Rods), not absolute rod saturation. Residual rod signals may indeed remain at our brightest adaptation point (‘b6’), yet are very small relative to the cones. Furthermore, accumulated noise at the level of cortex precludes the detection of smaller signals in the retina. Finally, there is the question of rod recovery dynamics at the lower light levels in our adaptation paradigm (b1–b5). These were played out in descending order following the brightest background condition (b6), and thus expected to be in a steadier regime of rod recovery’s temporal dynamics^25^.

Differences in rod saturation across studies may be further accounted for by two related experimental variables: 1) presence or absence of rod-cone interactions in the retina, and 2) recording location along the retinotopy. The overlapping spectral sensitivities of rods and cones makes it challenging to drive them independently. For this reason, studies have used mouse lines that lack functional cones. However, mice lacking cone function to assess rod saturation will not be subject to the substantial cone-rod crosstalk in the mouse^27,28^. Furthermore, more recent studies have shown that photoreceptor distributions and retinal circuits are more demarcated along the dorsoventral axis than originally thought^29–31^. As a result, rod-cone interactions may also affect rod saturation in mice with normal retinas, in a retinotopic-dependent fashion.

### Rod- vs. cone-mediated temporal receptive field properties in mouse visual cortex

Cone-mediated temporal dynamics in the retina are much faster than rods, and retinal ganglion cells respond to higher temporal frequencies when inputs originate from cones^4^. Therefore, a cone-induced tuning shift toward higher temporal frequencies by most V1 neurons (Fig. 5) is expected based on inheritance from responses at the output of the retina. However, the magnitude of the tuning shift in V1 cannot be directly inferred from retinal studies, as the cortex is known to attenuate higher temporal frequencies—there is low-pass filtering at the first synapse from LGN inputs^32^, but also between layer 4 and our recording location, layer 2/3^33^. This is consistent with the fact that the shift we observed in V1 is not quite as dramatic as what was observed in retinal ganglion cells by Wang et al.^4^ Another factor that may have influenced temporal frequency tuning in our preparation was anesthesia. V1 response dynamics in awake mice have much more rapid dynamics than anesthetized mice^ 34^, which may translate to greater responsiveness to higher temporal frequencies. Therefore, the cortical attenuation of rapid LGN dynamics may be alleviated in the awake prep, thus leading to a more dramatic shift to high temporal frequencies with cone-mediated vision than what has been observed in Fig. 5.

### Rod- vs. cone-mediated spatial receptive field properties in a rod dominated retina

We observed a 20% reduction in the size of V1 receptive fields (RFs) at photopic light levels, which may be attributed to inheritance of RF size adaptations in the retina. However, support is lacking for modulations of retinal ganglion cell (RGC) RF size. More specifically, studies report the absence of a systematic change in the size of the center-component of RGC RFs, albeit a partial disappearance of the surround in scotopic conditions^35–37^. Furthermore, if V1 were to inherit size-adaptions from the retina, one could expect a corresponding adaptation in SF tuning - viz. a shift toward higher spatial frequencies as RF sizes shrink - but this was not observed in our data. Instead, spatial frequency tuning was similar across light levels, and even changed subtly in the opposite direction of this model. To offer a descriptive explanation of these observations, we start with a 1D Gabor model of V1 RFs. If the sinewave frequency remains the same across states of light adaptation, but the Gaussian envelope widens during scotopic lighting, this would create the observed changes in our data—wider RFs and a slight shift in tuning toward higher SFs. We can only speculate on a mechanism that mediates a wider Gabor envelope; e.g. as a widening of cortical pooling from geniculate inputs. Alternatively, cone-mediated vision could yield relatively unbalanced ON and OFF input to V1 (e.g. due to a sparsely sampled cone mosaic), which would modulate V1 to lower SFs and smaller RFs, on average. One could model this scenario with adjacent difference-of-Gaussians (viz. ON-OFF), where the two Gaussian amplitudes are less balanced during the photopic state. 

Noise correlation in the retina may be another contributing factor to our combination of results on spatial tuning. It was recently shown that both the magnitude and spatial decay constant of pairwise noise correlations between RGCs is much higher during scotopic lighting^38^. Measured V1 RFs have the potential to widen under scotopic lighting due to these noise modulations in the retina. SF tuning with full-field gratings would nonetheless be invariant. To be clear, the proposed effect is an artifact due to insufficient averaging in our stimulus-triggered RFs, which is more pronounced when noise correlations are higher under scotopic lighting.

## Methods

### Animal preparation for surgery and imaging

All experiments were approved by the University of Texas at Austin’s Institutional Animal Care and Use Committee, which maintains AAALAC accreditation. All methods were carried out in accordance with relevant guidelines and regulations. When applicable, procedures were performed in accordance with ARRIVE guidelines. Mice of either sex from C57BL/6 and 129SV strains were used: 43 wild-type mice (C57BL/6; aged 2–5 months; 25 male), and 27 Gnat1^−/−^ mice (129SV; aged 2–5 months; 15 male). The Gnat1^−/−^ strain has a mutation in the rhodopsin gene, causing rod dysfunction. The Gnat1^−/−^ was originally generated on the BALB/c background^10^ in the laboratory of Dr. Janice Lem (Tufts University, Medford, MA, USA) and was donated for this study by Dr. Alapakkam P. Sampath (University of California, Los Angeles, CA, USA).

For all surgical procedures, mice were anesthetized with isoflurane (3% induction, 1–1.5% surgery), and given a pre-operative subcutaneous injection of analgesia (5 mg/kg Carprofen) and anti-inflammatory agent (Dexamethasone, 1%). Mice were kept warm with a water-regulated pump pad. Each mouse underwent two surgical procedures. The first was the mounting of a metal frame over the visual cortex using dental acrylic, which allowed for mechanical stability during imaging. The second was a craniotomy (4–4.5 mm in diameter) over V1 in the right hemisphere, virus injections, and a window implant (3-4 mm glass window). Surgical procedures were always performed on a separate day from functional imaging. On a day between the frame implant and virus injections, we identified the outline of the V1 border by measuring the retinotopic map with intrinsic signal imaging (ISI) through the intact bone^12,39^. This allowed for a more uniform distribution of virus across the V1 topography.

The virus (pAAV.Syn.GCaMP6f.WPRE.SV40, Addgene viral prep # 100,837-AAV1)^40^ was delivered using a Picospritzer III (Parker) or Nanoliter injector (WPI) to 2–3 sites in V1 with 0.25–0.5ul per site. Once the injections were complete, craniotomies were sealed using Vetbond and dental cement with dual-layered windows made from coverglass (4 mm glued to 5 mm, Warner Instruments), and covered between imaging sessions.

Common to all functional imaging procedures, isoflurane levels were titrated to maintain a lightly anesthetized state while the mouse laid on a water-circulating heat pad. However, mapping the V1 retinotopy using ISI is typically more tolerant to high isofluorane levels. For ISI, isoflurane levels were set to 0.25–0.8% (typically 0.5%). For two-photon and widefield calcium imaging, each mouse was given chlorprothixene (1.25 mg/kg) intramuscularly and isoflurane levels were adjusted to 0.25–0.5%. Silicone oil (12,500 cst) was periodically applied to the stimulated (left) eye to prevent any dryness or damage to the eye. Silicone oil allows optical transmission of near-UV (nUV) and visible wavelengths. Additionally, the unstimulated (right) eye was coated with Vaseline and covered with black tape during imaging.

### Imaging

Widefield images were captured using a Pco Panda 4.2 camera with Matlab’s image acquisition toolbox, at 10-to-20 frames/sec, and 2 × 2 binning. An X-Cite 110LED (Excelitas) was used for illumination. For (reflectance-based) intrinsic signal imaging, a longpass colored glass filter (590 nm) was placed over the light guides to illuminate the brain, and a bandpass filter (650 ± 25 nm) was on the camera lens. For widefield fluorescence GCaMP6f imaging, a GFP filter cube was placed on the camera lens.

Two-photon calcium imaging was performed with a Neurolabware microscope and Scanbox acquisition software. The scan rate varied between 10–15 frames/sec, scaling with the number of rows in the field-of-view. A Chameleon Ultra laser was set to 920 nm to excite GCaMP6f. A Nikon 16x (0.8NA 3 mm WD) or Olympus 10x (0.6NA 3 mm WD) objective lens was used for all imaging sessions. All cells were imaged between 150 and 350 um depth, which corresponds to layer 2/3 in the mouse.

### Visual stimuli

#### Setup overview

Two monochrome LED projectors (Keynote Photonics) with spectral peaks at 405 nm (nUV) and 525 nm (green) were used to generate spatio-temporally modulated stimuli that drive opsins in the mouse retina (Fig. 1a-d)^12^. The refresh rate was 60 Hz. The rear projector screen was made of Teflon, which provided a near-Lambertian surface (Fig. 1d). The two projectors were first independently mounted on an articulating platform to align the images as closely as possible, followed by a more refined software alignment consisting of a lateral shift and affine transformation. This software calibration procedure first entailed showing a grid of dots by the green projector, and a user-controlled mouse shown by the nUV projector. The user is prompted to “click” on each point in the grid, which is used to compute the transformation that aligns the projector images. Stimuli were coded using the Psychophysics Toolbox extension for Matlab^41,42^.

The mouse was positioned so that its left eye was vertically centered on the projector screen, and the perpendicular bisector from the mouse’s eye to the screen was between 8 and 10 cm. Next, the screen was angled at 30° from the mouse’s midline. The final constraint was to align the front edge of the screen to the mouse’s midline.

Image size and intensity vary with the distance between the projector and the Teflon screen. We selected a screen-to-projector distance that yielded a maximum image size just large enough to drive the majority of V1, while keeping intensity high. The screen size was 43 cm high × 23.5 cm wide, which gives approximately 135° × 105° of visual angle.

#### Measuring the uniformity of spectral radiance

Our experiments required visual stimuli that are calibrated for UV and visible wavelengths across a wide range of viewing angles, which ultimately requires an efficient diffuser of rear-projected nUV and visible light. We tested multiple commercial rear-projection films, but found that 0.01″ Teflon (McMaster-Carr)^19^ was unmatched in its optimization of our criteria. Criteria were based on uniformity of spectral radiance, across screen position and viewing angle. For each measurement, both green and nUV projectors showed a uniform image on the opposite side of the rear projection film as a PR655 spectroradiometer. The spectroradiometer was focused on the screen at 7 locations—center, top edge, bottom edge, and each corner (only center location measurement shown in Fig. 1d). At each location, a measurement was taken at three viewing angles relative to the planar screen, 30°, 60°, and 90°, totaling 21 measurements of spectral radiance. Ideally, the spectral radiance does not change across these 21 conditions. Figure 1d summarizes the results from the material we settled on (0.01″ Teflon) for the center location. The spectral radiance curves are virtually identical across viewing angles. Furthermore, the amplitude of the radiance curve across the various other locations and viewing angles (not shown) is also quite consistent. This uniformity in spectral radiance is far superior to our measurements of other commercial displays (a CRT or LCD monitor), other commercial rear projection materials, and thinner Teflon (0.005″). We would expect that the uniformity to be maintained for thicker Teflon, yet this would have the undesirable effect of attenuating the overall intensity of the image.

#### Stimulus A: Retinotopic mapping stimulus

The same drifting bar stimulus was used to map retinotopy, regardless of imaging modality. This method is an extension of the one used in Kalatsky and Stryker (2003)^11^, and described in detail in Marshel et al.^43^ To summarize, a periodic drifting bar on black background was modulated by a contrast-reversing checkerboard, and shown in four cardinal directions. The bar’s speed and width were dependent on screen location such that the temporal phase of cortical responses, at the stimulus frequency, could be directly mapped to altitude and azimuth coordinates in a spherical coordinate system. The speed of the bar was ~ 5°/s and its width was ~ 12°. The checkerboard squares were 5° wide and reversed contrast at a rate of 2 Hz. Both green and nUV projectors were shown at maximum contrast to produce the checkerboard. Each drift direction was shown at ~ 0.055 HZ for wide-field imaging and ~ 0.042 HZ for the 2-photon calcium imaging setup. There were 2-to-3 120 s trials, for each drift direction.

#### Stimulus B: Color tuning at graded light adaptation

With the goal of systematically varying rod saturation, a total of 6 different mean light levels were used across the experiments in this study (b1-b6), while keeping contrast the same (Figs. 1e, 2a). Each intensity was fixed for an entire block of drifting grating trials; prior to each, the eye was adapted for 10 min with a uniform screen of matched mid-point intensity. To quantify the graded transition from rod- to cone-mediated inputs, all 6 light levels were used, and the responses to M-isolating and S-isolating gratings (see below) were compared (Figs. 2–4). Furthermore, the spatial and temporal frequencies were kept constant across trials at 0.05 cyc/° and 1 cyc/sec, respectively, which are well represented in mouse V1^33,44^. Drift direction varied across trials: 0, 45, 90, 135, 180, 225, 270, and 315°. Each stimulus was shown for 3 s, flanked by 1 s of pre-stimulus and 1 s of post-stimulus blanks. The mid-point “gray level” was held constant throughout the entire adaptation and test stimuli.

#### S- and M-opsin isolating gratings

Green and nUV LED sinewaves were combined to produce contrast along the S- and M-isolating directions of cone-opsin space (Fig. 1f). As shown below, S-opsin and M-opsin contrast can be described as a function of the rear-projected spectral radiance of the nUV $(R_{UV} (\lambda ))$ and Green $(R_{G} (\lambda ))$ LEDs, the amplitude of their drifting sinewaves $(a_{UV} ,a_{G} )$, and the S- and M-opsin sensitivity functions ($(h_{S} (\lambda )$ and $h_{M} (\lambda ))$:

$$S\,contrast = \frac{{\mathop \sum \nolimits_{\lambda } h_{S} \left( \lambda \right)\left[ {a_{UV} R_{UV} \left( \lambda \right) + a_{G} R_{G} \left( \lambda \right)} \right]}}{{\mathop \sum \nolimits_{\lambda } h_{S} \left( \lambda \right)\left[ {R_{UV} \left( \lambda \right) + R_{G} \left( \lambda \right)} \right]}}$$

$$M\,contrast = \frac{{\mathop \sum \nolimits_{\lambda } h_{M} \left( \lambda \right)\left[ {a_{UV} R_{UV} \left( \lambda \right) + a_{G} R_{G} \left( \lambda \right)} \right]}}{{\mathop \sum \nolimits_{\lambda } h_{M} \left( \lambda \right)\left[ {R_{UV} \left( \lambda \right) + R_{G} \left( \lambda \right)} \right]}}$$

Spectral radiance was measured using a PhotoResearch PR655. Opsin sensitivity functions are taken from Govardovskii et al.^45^ To null either S- or M-opsin contrast, we utilized “silent substitution”^46^. M-isolating stimuli (“*S contrast*” = 0) can be achieved by limiting modulation to the Green LED $(a_{G} > 0)$, while keeping the nUV LED constant $(a_{UV} = 0)$. This is because $R_{G} \left( \lambda \right)$ and $h_{S} \left( \lambda \right)$ do not overlap (Fig. 1a,d). However, for S-isolating stimuli (“*M contrast*” = 0), we used the equations above to solve for $a_{UV}$ and $a_{G}$. The maximum possible S-opsin contrast for the S-isolating stimulus was 60%. Therefore, the M-isolating stimulus was also shown at 60% contrast. The same calibration procedure was used to generate M and S contrast for *Stimulus D* and *E,* described below.

#### Stimulus C: Spatio-temporal frequency tuning, at low and high light adaptation

Full-field drifting sinewave gratings at variable spatio-temporal frequency settings were used to study the spatio-temporal frequency characteristics of rod-dominated versus cone-dominated regimes in V1. To measure temporal frequency tuning, five temporal frequencies were shown: 0.5, 1, 2, 4, and 8 (cyc/s), all at a spatial frequency of 0.05 cyc/deg (Fig. 5a-f). Only one color was shown, which had 60% contrast along the S + M axis of cone-opsin space (Fig. 1f). One of eight drift directions, 45° apart, were shown on each trial. Each stimulus in the ensemble was shown 5 times, giving the following number of trials: 5 × 8 × 5 (*temporal frequency* x *drift direction* x *repeat*; 200 trials). Each stimulus was shown for 3 s, flanked by 1 s of pre-stimulus and 1 s post-stimulus blanks. This 200-trial stimulus block was shown at two different states of light adaptation, “b1” (dimmest) and “b5” (2nd brightest). Each block was preceded by 10 min of adaptation at the same gray level. Next, the spatial frequency stimulus used the same template—two 200-trial light adaptation blocks that varied spatial frequency as follows: 0.0125, 0.025, 0.05, 0.1, 0.2 cyc/deg (Fig. 6a-h). Temporal frequency was held at 1 cyc/sec.

#### Stimulus D: Spatio-temporal frequency tuning, with rod- and cone-isolating contrast

Here, spatial or temporal frequency tuning was measured in a single mesopic adaptation block that varied opsin contrast. In the temporal frequency version of this experiment (Fig. 5g-i), stimuli had one of 5 temporal frequencies [0.5 1 2 4 8 cyc/sec], 2 color contrasts [M- or S-isolating], 8 drift directions (45° intervals), and 1 spatial frequency (0.05 cyc/deg). In the spatial frequency version (Fig. 6i-l), stimuli had one of 5 spatial frequencies [0.0125 0.025 0.05 0.1 0.2 cyc/deg], 2 color contrasts [M- or S-isolating], 8 drift directions (45° intervals), and 1 temporal frequency (1 cyc/s). In each case, light levels were held constant at either ‘b5’ or ‘b4’, which place the retina in a mesopic state. Also, the stimulus referred to as “M-contrast” (above) is a rod-isolating contrast for neurons in the upper visual field in a mesopic state.

#### Stimulus E: Mapping receptive fields, with rod- and cone-isolating contrast

To measure the width of receptive fields along the vertical axis of visual space (Fig. 7), horizontal bars were flashed on the screen at varying vertical position and color. The four possible color directions of the bar in the S/M opsin plane were S-, S+, M-, and M+ . In the upper visual field, M-opsin contrast only drives rhodopsin. The bars were 6° wide in visual angle, had a square spatial profile, and were sampled at every 2.8°. Each bar was shown, static, for 0.75 s with pre- and post- blank period of 0.25 s each. The horizontal length of each bar extended from the left to right edge of the screen. Each stimulus combination of color and position was repeated 20–30 times, and signals were averaged for each.

### Eye dilation experiment and rod isomerization rate

In a subset of experiments, *Stimulus B* (above) was run before and after pharmacologically-induced pupil dilation (1% tropicamide) on the same day. To track the pupil diameter across experiments a CMOS camera with IR sensitivity (Allied Vision Manta G-235B) was mounted with a long working distance Zoom lens (Navitar 6000) and a 590 nm longpass filter. The camera and lens were just behind the mouse and pointed at a 5 mm angled mirror within the mouse’s field-of-view to capture the pupil images. During 2-photon imaging, the IR excitation transmitted through the head and out of the eye for a crisp image of pupil diameter (Fig. 4b). After each block of stimuli set to a given level of light intensity, a picture of the pupil was taken. Pupil diameter was measured along the axis that roughly appeared to yield the maximum length since the image was taken at an angle.

### Quantifying photoreceptor isomerization rates for each light level

To estimate the photoisomerization rates of rods and cones in our preparation, we start by converting the measured radiance from the display, in Watts/steradian/${\mu m}_{display}^{2}$, into irradiance at the retina, in Watts/$\mu m_{retina}^{2}$. The first step is to multiply the display radiance by the steradians at the pupil. Steradians is roughly the area of the pupil over the squared distance between the display and the retina, A_pupil_/D^2^_*display*_. Next, to convert area on the display, ${\mu m}_{display}^{2}$, to area on the retina, ${\mu m}_{retina}^{2}$, we divide by the squared magnification of the image formation*,* D^*2*^_*retina*_* /*D^*2*^_*display*_, where D_*retina*_ is the diameter of the retina and D_*display*_ is the distance to the display. To simplify, D^2^_*display*_ cancels out, and we can just multiply the original radiance measurement by A_pupil_/D^*2*^_*retina*_ to get irradiance, in ${\text{Watts}}/\upmu {\text{m}}_{retina}^{2}$. The irradiance values are sampled at discrete wavelengths, $\Delta \lambda$, which gives units of ${\text{Watts}}/{\mu m}_{retina}^{2} /\Delta \lambda$. Finally, we scale by the transmittance at each wavelength, $T\left( \lambda \right)$, to compute the final retinal irradiance. We used a model for $T\left( \lambda \right)$ through the mouse lens, given in Lei and Yao^47^, which attenuates by 22.5% at 380 nm, and drops monotonically to 8.0% attenuation at 700 nm. To formalize, we convert radiance off the display, $^{\prime}Rad\left( \lambda \right)^{\prime},{ }$ into irradiance at the retina, $^{\prime}I\left( \lambda \right)^{\prime}$ as follows:

$$I\left( \lambda \right) = T\left( \lambda \right)*Rad\left( \lambda \right)*{\text{A}}_{pupil} /{\text{D}}_{retina}^{2} ;\quad {\text{Retinal irradiance}}\;{\text{(Watts}}/{\mu m}_{retina}^{2} /\Delta \lambda ).$$

Next, convert Joules to quanta

$$I_{Q} \left( \lambda \right) = I\left( \lambda \right)*\lambda/(c *h);\quad {\text{photons/s/mm}}^{2} /\Delta \lambda ;\quad c = {\text{speed of light,}}\;h = {\hbox{Planck's}}\;{\text{constant}}$$

To compute isomerization rate, $R^{*} /receptor/s$, we take the dot-product between the quantal retinal irradiance, $I_{Q} \left( \lambda \right)$, and the absorption spectrum, $a_{c} \left( \lambda \right),$ and scale by the sample period of the spectrum $\Delta \lambda$:

$$R^{*} /receptor/s = \Delta \lambda *\Sigma_{\lambda } I_{Q} \left( \lambda \right)a_{c} \left( \lambda \right);$$

To get $a_{c} \left( \lambda \right)$ in μm^2^, we started with the unitless absorption spectra of rods, M-opsin, and S-opsin from Govardovskii et al.^45^ and then scaled by the end-on collection area at the peak wavelength which may be approximated as near 1 and 0.85 μm^2^ for cones and rods, respectively^13^. To get the final isomerization rate in a given experiment, we scale by the corresponding projector intensity (e.g. buffer value), relative to the values obtained from the initial measurement of $Rad\left( \lambda \right)$. This gives the following range of isomerization rates, across the 6 light adaptation levels (Fig. 2a), for each opsin: M-opsin = [13.4 K 430 K], S-opsin = [2.75 K 88 K], and rhodopsin = [12 K 382 K].

### Quantifying photoreceptor isomerization rates as a function of solar elevation

A recent study measured solar irradiance in a rural setting, in absolute power units, with varying solar elevation^8^. Their calibrated units in Watts/m^2^ allow us to quantify isomerization rates in the mouse retina. For the nighttime irradiance, we used the same spectral profiles given in Spitschan et. al., as these contained values into UV. However, their daylight measurements did not contain the UV part of the spectrum, which is important for computing isomerization rates in the rodent. In turn, we used the unitless CIE D65 standard for daylight irradiance, which does include UV values, and scaled it by the Spitschan et. al. irradiance measurements at 450 nm. Spitschan et. al. found that the biggest deviations from the CIE D65 standard occur at lower solar elevations, which validates the use of the CIE D65 standard at higher solar elevations. In the equation below, $I_{outdoor} (\lambda |\emptyset )$ is the spectral irradiance in W/m^2^ at discrete solar elevations, $\emptyset$. To obtain the spectrum at each $\emptyset$, we took the average within a range of elevations, and across the medley variables that can affect it on a given day^8^. Next, the outdoor irradiance was converted into radiance at the retina by multiplying by the surface area of the pupil, A_pupil_, over the surface area of the retina, A_retina_^48^.

$$I\left( {\lambda |\emptyset } \right) = T\left( \lambda \right)*I_{outdoor} (\lambda |\emptyset )*{\text{A}}_{{{\text{pupil}}}} /{\text{A}}_{{{\text{retina}}}} ;\quad {\text{Retinal}}\;{\text{irradiance}}\;({\text{Watts}}/{\mu m}_{retina}^{2} /\Delta \lambda ).$$

Then, retinal irradiance is converted into isomerization rates, using the same manipulations as above for the display calculations. But now, it is expressed as a function of solar elevation:

$$R^{*} \left( \emptyset \right)/receptor/s = \Delta \lambda *\Sigma_{\lambda } I_{Q} \left( {\lambda |\emptyset } \right)a_{c} \left( \lambda \right).$$

In Fig. 4j, this is plotted for the case of $a_{c} \left( \lambda \right)$ being the rhodopsin sensitivity function, at three values of A_pupil_.

### Data analysis

#### Widefield imaging

Widefield images were captured to measure responses to two stimuli. The first was retinotopy (*Stimulus A*). The preferred phase at each pixel, at the frequency of the drifting bar, was computed for each of the four drift directions. The opposing directions (e.g. up and down) were then combined to yield retinotopic position as described in Kalatsky and Styker^11^. This gave a clear boundary of V1 that could be manually outlined. The second stimulus with widefield was used to measure color tuning at graded light adaptation (*Stimulus B*). On each trial, the time course of each pixel was normalized by a 400 ms blank period preceding grating onset, to yield a time course of ∆F/F (i.e. % response above the preceding baseline). Next, the mean was taken from 100 to 3100 ms after stimulus onset of the 3000 ms stimulus. We then averaged over 8 drift directions and 5 repeats to get the mean response of each pixel to the M-isolating stimulus and S-isolating stimulus. Finally, the map of $M_{pref}$ was computed as described in Eq. 1 below.

#### Generating neuronal tuning curves from two-photon movies

Neurons were identified using the local cross-correlation image, whereby the time course of each pixel was cross correlated with the weighted sum of its neighbors^49^. This is given as

$$LocalX\left( {x,y} \right) = \mathop \sum \limits_{t} F\left( {x,y,t} \right)\left[ {F\left( {x,y,t} \right) \otimes DoG\left( {x,y} \right)} \right]$$

where $F\left( {x,y,t} \right)$ are the fluorescence values at each pixel and timepoint, $DoG\left( {x,y} \right)$ is a difference-of-Gaussian image, and $\otimes$ indicates convolution in *x* and *y*. The DoG rewards pixels that are correlated in time with their immediate neighborhood, but also penalizes them if they are correlated in time with the broader surround. The central Gaussian of the DoG was near the size of a neuron body (σ = 3 µm), whereas the outer “suppressive” Gaussian had σ = 20 µm to capture the surrounding neuropil. The integral of each Gaussian was normalized. Including the suppressive surround in the weighting function yields brighter and more localized puncta in the resultant image. That is, groups of pixels about the size of a cell body could be more clearly disambiguated from the surround, which is important for the subsequent manual selection of cells. The puncta were manually selected using a “point-and-click” GUI. After clicking on a location, a local threshold was applied to $LocalX$ in order to identify the cell ROI. The pixels inside the ROI were then averaged to give the time course of each cell.

On each trial, a neuron’s raw fluorescence timecourse was converted into units of *∆F/F*. Specifically, *∆F/F* = [*F*(*t*)-*p*]/*p*, where *F*(*t*) is the raw signal and *p* is the mean fluorescence during a blank screen preceding the trial. The mean of *∆F/F* was then taken during the response. Most stimuli used in this study consisted of trials with drifting sine wave gratings presented for 3 s (*Stimulus B-D*), in which case *p* was taken over a 1000 ms window, and the response mean was taken between 500 to 3250 ms after stimulus onset. In the case of the static flashed bars (*Stimulus E*), the window for *p* was 250 ms, and the trial integral was taken between 250 and 850 ms. For *Stimulus A*, *∆F/F* was not calculated since we were only interested in the phase.

#### Model of photoreceptor inputs vs. light intensity and retinotopy

At each of 6 background light levels (see *Stimulus B*), we computed each neuron’s color preference metric defined below, where *F*_*s*_ and *F*_*m*_ are the mean *∆F/F* response to the S- and M-isolating stimuli.

1 $$M_{{{\text{pref}}}} = \frac{{F_{m} }}{{F_{s} + F_{m} }}\quad {\text{Color}}\;{\text{preference}}$$

In general, the rods are not fully saturated, so a neuron’s response to each color direction, *F*_*s*_ and *F*_*m*_, is a function of input from both rods and cones, which we represent as a linear combination below.

2 $$F_{s} \left( {b,v} \right) = w_{s} \left( v \right)s_{s} + w_{r} \left( b \right)r_{s} \quad {\text{Response}}\;{\text{to}}\;{\text{S - opsin}}\;{\text{grating}}$$

3 $$F_{m} \left( {b,v} \right) = w_{m} \left( v \right)m_{m} + w_{r} \left( b \right)r_{m} \quad {\text{Response}}\;{\text{to}}\;{\text{M - opsin}}\;{\text{grating}}$$

$w_{s} \left( v \right)$ and $w_{m} \left( v \right)$ are cone S- an M-opsin input weight as a function of vertical retinotopy, *v*. $w_{r} \left( b \right)$ is the rod input weight, as a function of rod isomerization rate, ${10}^{3} \;{\text{R}}^{*} {\text{/rod/s}}$, denoted *b*. Each opsin weighting function is unitless but scaled relative to each other. For instance, under low light levels where rods dominate, then $w_{r} \gg w_{s}$. Also, the sum of cone opsin inputs is unity across the retinotopy; i.e. $w_{s} \left( v \right) = 1 - w_{m} \left( v \right)$. To get the response contribution of each opsin, the weighting function is scaled by a stimulus contrast; *s*_*s*_ is the S-opsin contrast for the S-isolating stimulus (~ 0.6), and *m*_*m*_ is the M-opsin contrast of the M-isolating stimulus (~ 0.6). *r*_*s*_and *r*_*m*_ are the rod contrasts to the S- and M-isolating stimuli. Plugging Eqs. (2) and (3) into Eq. (1) gives

4 $$M_{{{\text{pref}}}} \left( {b,v} \right) = \frac{{w_{m} \left( v \right)m_{m} + w_{r} \left( b \right)r_{m} }}{{w_{s} \left( v \right)s_{s} + w_{r} \left( b \right)r_{s} + w_{m} \left( v \right)m_{m} + w_{r} \left( b \right)r_{m} }}\quad {\text{Model}}\;{\text{of}}\;{\text{color}}\;{\text{preference}}$$

Unlike rod weights in this model, cone weights do not depend on the light level. This is based on our measurements in Gnat1^−/−^ mice, where responses were roughly constant across the range of light levels, *b* (Fig. 3a-b). Also, the lowest light levels in our experiments place the isomerization rate of each cone just above 5 K R*/cone/s, which is above the dark noise according to prior studies, at which point Weber adaptation takes over^13,14,50^. Lastly, whereas rod weights are assumed independent of the retinotopy since the mouse lacks a fovea, the balance of M and S-opsin weights are dependent on the vertical retinotopy^12^.

Finally, Eq. (4) can be simplified to help with subsequent manipulations and model fitting. Since rhodopsin and M-opsin have very similar sensitivity functions, we can assume that rod contrast to the M-isolating stimulus is much greater than rod contrast to the S-isolating stimulus. Or $r_{m} \gg r_{s}$. After plugging in $s_{s} = m_{m}$ (i.e. equated cone-opsin contrast) and $w_{m} \left( v \right) = 1 - w_{s} \left( v \right)$, this simplifies to

5 $$M_{{{\text{pref}}}} \left( {b,v} \right) \sim 1 - \frac{{w_{s} \left( v \right)s_{s} }}{{s_{s} + w_{r} \left( b \right)r_{m} }}\quad {\text{Simplified}}\;{\text{model}}\;{\text{of}}\;{\text{color}}\;{\text{preference}}$$

Near rod saturating light levels, $w_{r} (b) \ll w_{s} (v)$, so that $M_{{{\text{pref}}}} \left( {b,v} \right)$ approaches $1 - w_{s} \left( v \right) = w_{m} \left( v \right)$. At the opposite extreme, when light levels are low, $w_{r} \left( b \right) \gg w_{s} \left( v \right)$, and $M_{{{\text{pref}}}} \left( {b,v} \right)$ approaches 1. $M_{{{\text{pref}}}} \left( {b,v} \right)$ is the measured variable and $w_{s} \left( v \right)$ and $w_{r} \left( b \right)$ are unknowns. Equation (5) has the convenient feature of separability along *v* and *b*. As described below, we first fit the data along *b*, and then *v*.

#### Calculating rod input vs. R*/rod/sec

To simplify the calculation of rod input, $w_{r} \left( b \right)$, we limited data points to the upper visual field (> 30°) where the S-opsin contribution from the cones is near 100% (Wang et al.^4^ ; i.e. $w_{s} \left( {v > 30^\circ } \right) = 1$.

6 $$M_{{{\text{pref}}}} \left( {b,v > 30^\circ } \right) = 1 - \frac{{s_{s} }}{{s_{s} + w_{r} \left( b \right)r_{m} }}\quad {\text{Color}}\;{\text{preference}}\;{\text{in}}\;{\text{upper}}\;{\text{fields}}$$

We then fit $M_{pref} \left( {b,v > 30^\circ } \right) = e^{{ - \alpha b^{\gamma } }}$ to the data, which gave $\alpha$ = 0.072 and $\gamma$ = 0.586. Again, ‘*b*’ is in units of 10^3^ R*/rod/sec. Setting $e^{{ - \alpha b^{\gamma } }}$ equal to Eq. (6), then plugging in the rod and cone opsin contrast values, $r_{m} \sim s_{s} = 0.6$, and finally solving for $w_{r} \left( b \right)$ gives

7 $$w_{r} \left( b \right) = 1/[e^{{0.07b^{0.59} }} - 1]\quad {\text{Relative}}\;{\text{rod}}\;{\text{input}}\;{\text{vs}}\;10^{3} {\text{R*/rod/sec}}$$

$w_{r} \left( b \right)$ is unitless and scaled relative to the cone opsin weights. A more meaningful model parameter is obtained by calculating the rod weight, relative to the total rod and cone weight:

8 $$\% {\text{Rod}}\left( b \right) = \frac{{w_{r} \left( b \right)}}{{w_{r} \left( b \right) + w_{s} \left( v \right) + w_{m} \left( v \right)}} = \frac{{w_{r} \left( b \right)}}{{w_{r} \left( b \right) + 1}} = e^{{ - 0.07b^{0.59} }} \quad {\text{\% Rod}}\;{\text{input,}}\;{\text{relative}}\;{\text{to}}\;{\text{the}}\;{\text{cones}}$$

Importantly, Eqs. (6) and (8) are equivalent: $M_{{{\text{pref}}}} \left( {b,v > 30^\circ } \right) = \% {\text{Rod}}\left( b \right)$. That is, the measured color preference in the upper visual field is also a measure of the rod preference. Also, we have expressed $\% {\text{Rod}}\left( {b, v > 30} \right)$ as $\% {\text{Rod}}\left( b \right)$, which assumes that rod saturation is constant if irradiance is constant.

#### Calculating cone opsin balance vs. retinotopy

Finally, we fit a model to $w_{m} \left( v \right) = 1 - w_{s} \left( v \right)$, which is the balance of M- and S-cone opsin at each location of the vertical retinotopy, independent of the rods. Coming back to Eq. 5, which is the model of the observed variable $M_{{{\text{pref}}}} \left( {b,v} \right)$, we can solve for $w_{m} \left( v \right)$ at each data point since $w_{r} \left( b \right)$ was solved for in Eq. 7. Then, we fit the following sigmoid (Fig. 3f, red).

9 $$w_{m} \left( v \right) = \frac{1}{{1 + e^{{0.192\left( {v - 14} \right)}} }}\quad {\text{Balance}}\;{\text{of}}\;{\text{cone}}\;{\text{opsin}}\;{\text{vs}}{.}\;{\text{retinotopy}}$$

#### Quantifying spatio-temporal tuning

To get the spatial or temporal frequency tuning curve of each cell, at each background level (Stimulus C) or color (Stimulus D), we averaged responses over repeats, and then drift direction. Next, a Gaussian function with a domain of log_2_(spatial frequency) or log_2_(temporal frequency) was fit in order to quantify tuning curves. For both temporal and spatial frequency tuning, center-of-mass and high-pass cutoff were calculated from the fit. For only spatial frequency, the peak location of the fit was also used.

Center-of-mass was determined in the logarithmic domain as ${\text{CoM}}_{{{\text{log}}}} = \mathop \sum \nolimits_{f} G\left( {\log_{2} \left( f \right)} \right){\text{*log}}_{2} \left( f \right) / \mathop \sum \nolimits_{f} G\left( {\log_{2} \left( f \right)} \right)$, where *G*()is the Gaussian fit, and *f* is the stimulus domain in cyc/deg or deg/sec. ${\text{CoM}}_{{{\text{log}}}}$ is not necessarily the peak location of the Gaussian because the sum over *f* is limited to the stimulus domain. Then, ${\text{CoM}}_{{{\text{log}}}}$ was converted into the same units as *f* by taking $2^{{{\text{CoM}}_{{{\text{log}}}} }}$. Next, peak location and high-pass cutoff were both determined from a finely sampled version of the Gaussian fit. Note that the peak location differed from the parameter μ in the Gaussian fit when the tuning was low-pass. High-pass cut-off frequency was taken at 70% of the peak’s height, to the right of the peak.

#### Two-photon data yield

All data analysis followed an initial preprocessing pipeline for quality control. To be included in the analysis, each neuron had to cross a response threshold for two stimuli: (1) Its mean ∆F/F response in the stimulus window of the sinewave grating stimulus-of-interest (i.e. *Stimuli B, C, D,* or *E*) and (2) its amplitude of response at the stimulus frequency of the drifting bar stimulus, which was used to map retinotopy (*Stimulus A*). A cell was excluded if (1) it did not have a mean ∆F/F > 0.05 for any stimulus within the ensemble of episodic stimuli, OR (2) if the magnitude of the Fourier amplitude at the stimulus frequency did not have a peak sufficiently greater than the noise—specifically, unreliable activity was identified based on the ratio of the first harmonic (F1) magnitude over the mean of the Fourier magnitudes at neighboring frequencies (between 0 and 0.14 Hz). If this ratio was less than 5 for any drift direction, it was excluded from subsequent analyses.

For experiments that required fitting a Gaussian curve (see “*Quantifying spatio-temporal tuning*”), an additional screening was applied based on the variance accounted by the fit. A threshold of 65% and 45% was applied to WT and Gnat1^−/−^, respectively.

The majority of the animals used in this study generated data for a single figure. Of the 43 wild-type mice, 7 were used for the pupil experiment (Fig. 4), 11 for the graded adaptation experiment (Figs. 2, 3), 6 for the temporal frequency experiment (Fig. 5), 14 for the spatial frequency experiment (Fig. 6), and 12 for the receptive field size experiment (Fig. 7). Of the 27 Gnat1^−/−^ mice, 3 were used for the graded light adaptation (Fig. 3a,b), 12 for the temporal frequency experiment (Fig. 5), and 12 for spatial frequency experiment (Fig. 6).

The following summarizes WT cell survivability based on the above threshold criteria applied to the analyses within each figure sub-panel. Pupil experiment (Fig. 4b,c): 386/619 cells. Temporal frequency experiment (Fig. 5a-c): 235/662 cells. (Fig. 5g-i): 218/334. Spatial frequency experiment (Fig. 6a-d): 250/1154 cells. (Fig. 6i-l): 361/818 at 44.13%. Receptive field size mapping (Fig. 7): 108/1082 cells. For the graded light adaptation experiments in 11 WTs (Figs. 2, 3), the yield varied slightly across the different states of adaptation, “b1-b6”. In 2 of the 11 animals, b6 was not run. For b6, the yield was 666/1014 cells. For b1-b5 (11 animals), the yield ranged from 773/1252 at b2, up to 791/1252 at b5. The Gnat1^−/−^ yield was as follows: Temporal frequency experiment (Fig. 5d-f) 244/1218 cells at 20.03%. Spatial frequency experiment (Fig. 6e-h): 72/838 cells at 8.59%. For the graded light adaptation (Fig. 3a,b), the yield ranged from 94/215 at b1, up to 102/215 at b6.

## Data Availability

Data from this study is available upon reasonable request.

## Code availability

Matlab code contributing to this study is available upon reasonable request.
