# Author Correction: Variations in photoreceptor throughput to mouse visual cortex and the unique effects on tuning

**DOI:** 10.1038/s41598-023-39131-4

**Published:** 2023-07-24

**Authors:** I. Rhim, G. Coello‑Reyes, I. Nauhaus

**Affiliations:** 1 Department of Psychology, University of Texas At Austin, 108 E. Dean Keeton, Austin, TX 78712 USA; 2 Department of Neuroscience, University of Texas At Austin, 1 University Station, Stop C7000, Austin, TX 78712 USA; 3 Center for Perceptual Systems, University of Texas At Austin, 108 E. Dean Keeton, Austin, TX 78712 USA

Correction to: *Scientific Reports* 10.1038/s41598-021-90650-4, published online 07 June 2021

The original version of this Article contained an error in the Methods section, where the equation to convert from Watts to photons was incorrect.

Under the sub-heading ‘Quantifying photoreceptor isomerization rates for each light level’

$$IQ\left(\lambda \right)=I\left(\lambda \right)*\lambda *c/h$$

now reads:

$$IQ\left(\lambda \right)=I\left(\lambda \right)*\lambda /(c*h)$$

The original Article has been corrected.

